# An antibody-drug conjugate targeting soluble and membrane-bound TGFα is effective against pancreatic tumors

**DOI:** 10.1186/s13046-025-03421-8

**Published:** 2025-05-23

**Authors:** Inés Romero-Pérez, Juan Carlos Montero, Mónica Redondo-Puente, María del Carmen Gómez-García, Mireia Morell-Ginestà, Gabriel Capellá, Atanasio Pandiella

**Affiliations:** 1 Instituto de Biología Molecular y Celular del Cáncer- CSIC and CIBERONC, Campus Miguel de Unamuno, Salamanca, 37007 Spain; 2 Department of Pathologic Anatomy and IBSAL, Salamanca, Spain; 3 Hereditary Cancer Program, Catalan Institute of Oncology, Institut d’Investigació Biomèdica de Bellvitge- IDIBELL-ONCOBELL, L’Hospitalet de Llobregat, Barcelona, 08908 Spain; 4 Centro de Investigación Biomédica en Red Cáncer (CIBERONC), Madrid, Spain

## Abstract

**Background:**

Pancreatic cancer is one of the most difficult to treat neoplasias. Because of that, the prognosis of the disease is dismal, and identification of novel therapeutic approaches is needed. This study investigates the role of transforming growth factor-alpha (TGFα) in pancreatic cancer and its potential as a therapeutic target.

**Methods:**

Using in silico platforms, it was confirmed that *TGFA*, the gene encoding TGFα, is significantly overexpressed in pancreatic adenocarcinomas relative to normal pancreatic tissues. In patient-derived xenografts as well as in pancreatic cancer cell lines, multiple molecular forms of TGFα were identified, including the transmembrane TGFα precursor (proTGFα) and the soluble 6 kDa mature form. Functional assays using RNA interference and CRISPR/Cas9 demonstrated that *TGFA* knockdown significantly impaired cell proliferation, reinforcing the critical role of TGFα in driving tumor growth. The therapeutic potential of targeting TGFα was evaluated through the development of two monoclonal antibodies (5F1 and 16B10) specific for TGFα.

**Results:**

These antibodies effectively bound to proTGFα-expressing cells, with minimal off-target effects in *TGFA*-knockout cell lines. When conjugated to cytotoxic agents such as MMAF, the resulting antibody-drug conjugates (ADCs) exhibited potent antiproliferative activity, significantly reducing the viability of TGFα-expressing pancreatic cancer cells. Mechanistic studies revealed that MMAF-loaded ADCs induced G2/M cell cycle arrest, with markers of mitotic disruption evident in treated cells. In vivo, the TGFα-targeting ADCs elicited substantial tumor regression in murine models of pancreatic cancer, whereas the unconjugated antibodies merely stabilized tumor growth.

**Conclusions:**

These findings highlight TGFα as a promising therapeutic target in pancreatic cancer, supporting further preclinical and clinical development of TGFα-directed ADCs.

**Supplementary Information:**

The online version contains supplementary material available at 10.1186/s13046-025-03421-8.

## Introduction

Pancreatic tumors are one of the most lethal forms of cancer [1]. They represent about 3% of all cancers in the United States, but account for approximately 7% of all cancer-related deaths. Pancreatic ductal adenocarcinomas (PDAC) are the most frequent ones, reaching 95% of all exocrine pancreatic neoplasias [2]. Multiple risk factors contribute to pancreatic cancer [3]. Age is a major determinant, with risk increasing significantly after the age of 60, while cigarette smoking is considered one of the strongest environmental risk factors. Other factors include chronic pancreatitis, diabetes, obesity, a family history of pancreatic cancer, and certain hereditary genetic mutations, such as those found in *BRCA1* and *BRCA2* [3, 4]. The treatment options for pancreatic cancer depend largely on the stage at which it is diagnosed. Surgery is the only potential curative option, but only 20% of patients are eligible due to late detection [3]. For unresectable or metastatic cases, chemotherapy (e.g., FOLFIRINOX, gemcitabine) and radiation therapy are the mainstays of treatment. However, the 5-year survival rate remains dismal, at less than 10% [1], mainly due to late diagnosis, the aggressive characteristics of the disease, and the poor therapeutic options once the disease has disseminated. Because of this, novel effective therapeutic strategies are needed.

Transforming growth factor α (TGFα) is a 6 kDa protein that belongs to the epidermal growth factor (EGF) family of polypeptide growth factors [5]. TGFα is synthesized as a larger transmembrane protein of 18 kDa, termed proTGFα, that undergoes several posttranslational modifications to generate a 17 kDa membrane-bound proTGFα [6, 7]. This form may be subjected to proteolytic cleavage by cell surface proteases, generating soluble TGFα and a cell-bound 15 kDa fragment that includes a short stretch of extracellular aminoacids, the transmembrane region and the cytoplasmic domain [7, 8]. TGFα acts by binding to the epidermal growth factor receptor (EGFR) [9]. Such interaction facilitates receptor oligomerization [10] which is required to initiate signaling, which includes activation of pathways, such as the MAPK and PI3K routes, which promote cellular proliferation [11].

Certain studies have linked increased expression of TGFα to the pathophysiology of pancreatic cancer. Thus, transgenic mice expressing high levels of the protein develop hyperplastic and metaplastic changes of the pancreas, together with an increase in the size of the organ [12–14]. In humans, PDACs have been reported to express high levels of both TGFα and EGFR [15–17]. Continuous engagement of this signaling loop may contribute to enhancing the proliferative capacity of pancreatic tumor cells, prevent apoptosis, and promote resistance to chemotherapy. Moreover, the role of TGFα in pancreatic cancer progression is not only restricted to enhancing cell proliferation but also involves its effect on the tumor microenvironment. TGFα contributes to the desmoplastic reaction (fibrotic tissue formation) observed in pancreatic tumors, which creates a dense stroma that makes the tumor more resistant to therapeutic interventions [18–21].

The fact that TGFα may have a role in the development of pancreatic cancer, together with its structural properties as a membrane-bound protein, opens the possibility of targeting this growth factor with therapeutic purposes. A potential strategy would be the use of antibodies against TGFα, that may not only recognize the soluble factor, but also its membrane-bound form. These antibodies against mature TGFα could neutralize its growth promoting activities, but may also bind transmembrane proTGFα, offering the possibility of using the latter as a potential antibody-drug conjugate (ADC) target.

Antibody-drug conjugates are sophisticated versions of antibodies, formed by three components: a monoclonal antibody against a cell surface protein, a cytotoxic drug, and a linker used to chemically connect the antibody to the cytotoxic [22, 23]. ADCs bind to the extracellular region of a transmembrane protein and are then internalized to lysosomes, a site in which they undergo proteolytic cleavage [24]. That process releases the cytotoxic payload that can then be transported to the cytosol to finally reach the cellular target, usually microtubules, DNA or proteins related to DNA stability. Several ADCs have entered the oncology clinic and their use is on the rising, with more than 210 ongoing clinical trials [25].

Here, we demonstrate that expression of TGFα facilitates proliferation of pancreatic cancer cells. Moreover, the potential therapeutic value of antibodies and ADCs prepared to recognize both mature TGFα as well as proTGFα has been explored. We show that those ADCs exert potent and specific antitumoral action on human pancreatic cancer cell lines both in vitro and when injected in mice.

## Materials and methods

### Reagents and antibodies

Cell culture media, fetal bovine serum (FBS), penicillin/streptomycin and trypsin-EDTA were purchased from Life Technologies (Carlsbad, CA, USA). Protein A-Sepharose^®^ and GammaBind G-Sepharose^®^ were from GE Healthcare (Little Chalfont, UK). Immobilon^®^ PVDF membranes were purchased from Millipore Corporation (Billerica, Massachusetts, USA), and autoradiography films were from Agfa-Gevaert (Mortsel, Belgium). 6-diamidino-2-phenylindole (DAPI) and puromycin were from Sigma-Aldrich (St Louis, MO, USA). Polyethyleneimene (PEI) reagent was from Polysciences (Hirschber an der Bergstrasse, Germany). Lentiviral vectors and specific plasmids were supplied by Cultek (Madrid, Spain), Addgene (Cambridge, Massachusetts, USA), GenScript, Thermo Fisher Scientific or Sigma-Aldrich. The Safe & Easy Toxin (SET™) was from Levena Biopharma (San Diego, CA, USA). Other general chemicals were from Roche Biochemicals (St. Luis, MO, USA), Merck (Darmstadt, Germany) and USB Corporation (Cleveland, OH, USA).

The rabbit polyclonal anti-calnexin antibody (SPA-860-F) was from Stressgen Biotechnologies Corporation (British Columbia, Canada). Horseradish peroxidase conjugates of anti-rabbit or anti-mouse immunoglobulin G were from Bio-Rad Laboratories (Hercules, USA) and GE Healthcare Life Sciences (Piscataway, NJ, USA), respectively. Antibodies against GAPDH (sc-166574) and pTyr99 (sc-7020) were from Santa Cruz Biotechnology (Santa Cruz, CA, USA). The anti-EGFR and anti-proTGFα polyclonal antibodies have been described previously [26, 27]. The neutralizing goat anti-human TGFα polyclonal antibody was from R&D Systems (Minneapolis, MN, USA). The anti phospho-H3 (S10) (#06-570) was from Merck Millipore. Antibody against LAMP-1 (#9091) was from Cell Signaling Technologies (Beverly, MA, USA). Antibody against BubR1 (#612503) was from BD transduction Laboratories (San Jose, CA, USA). The anti-MMAF (#62538) antibody was from Invitrogen. The anti-DM1, anti-DXd and R163 antibodies have been made in our laboratory. The anti-TGFα 5F1 and 16B10 monoclonal antibodies were made by BIOTEM (Apprieu, France).

### Cell culture, cell proliferation, cell cycle, and apoptosis assays

All cell lines were cultured at 37ºC in a humidified atmosphere in the presence of 5% CO2 and 95% air in Dulbecco’s modified Eagle’s medium (DMEM), supplemented with 10% FBS containing high glucose (4500 mg/liter) and antibiotics (penicillin 100 U/ml, streptomycin 100 µg/ml). Cells were tested for authenticity by STR at the Hematology Service of the University of Salamanca. NP29 and NP31 cells were obtained from patient samples by Dr. Gabriel Capellá. Cell proliferation experiments were conducted using a Z1 Coulter Particle Counter as described previously [28, 29]. Apoptosis and cell cycle analyses were performed as described [30]. The *KRAS* mutation status of genomic DNA isolated using TRIzol (Thermo Fisher Scientific) from Capan-1, IMIM-PC1, IMIM-PC2, SKPC1, NP29, and NP31 cell lines was analyzed with the *KRAS* Mutation Test v2 (LSR) on the Cobas^®^ platform (Roche Diagnostics) by PCR using the Cobas^®^ z 480 analyzer (Roche). The resulting data are presented in Supplementary Table 1.

### Gene knockdown/knockout and reconstitution of TGFA protein levels

To modify the expression levels of *TGFA* in pancreatic cancer cell lines, cells were infected with different plasmids using lenti or retrovirus. For the knockdown or knockout of *TGFA*, we proceeded to produce lentivirus. For this, we co-transfected 4 µg of pDMLg/RRE, pRSV-Rev and pMD2.G plasmids (Addgene, Cambridge, MA, USA), with 8 µg of the shRNA_*TGFA* (sequences #75-AAC ACA ATA CCC AGA GCG AAC or #77-AGC ACA CAT GTG ATG ATA AGG), the control vector pLKO.1_shRNA, or 8 µg of a *pool* with 3 different CRISPR/Cas9 plasmids (pLentiCRISPRv2_*TGFA*_cRNA1, 2 or 3 (GenScript)), into HEK293T cells using JetPEI^®^ reagent (Polyplus transfection, Illkirch, France), following the manufacturer’s instructions. Twenty-four hours later, HEK293T medium was replaced with fresh medium and 48 h after the co-transfection, the medium containing the lentiviral particles was collected, filtered with 0.45 μm PVDF filters, and utilized to infect cells after the addition of 6 µg/mL polybrene (Sigma-Aldrich, St. Louis, MO, USA). Seventy-two hours later, cells were selected with 3–6 µg/µL of puromycin, depending on the cell line, for another 72 h. To establish new cell lines with reconstituted *TGFA* expression, *TGFA*-knockout cells were infected with retrovirus generated using the following plasmids: 6 µg of pLZR_hTGFα, 5 µg of pMDG-VSV-G and 10 µg of pNGVL-MLV-gag-pol.

All infected cell lines for both knockout and reconstitution of *TGFA* expression were maintained for several weeks without puromycin. Single *TGFA* KO or reconstituted cells were selected by single-cell sorting by flow cytometry. To do this, cells were treated with 10 nM of the anti-TGFα polyclonal antibody (R&D) for 20 min at 37 °C. After this, cells were trypsinized, collected, centrifuged at 1,200 rpm for 5 min, and resuspended in PBS + 2% BSA. Subsequently, cells were incubated with an anti-goat Cy3 antibody (1:1.000, Abcam) for 30 min in agitation in the dark at room temperature. After this time, cells were washed three times with PBS + 2% BSA and resuspended in the same buffer. Finally, cell-surface expression levels of TGFα were analyzed using a cytometer (BD FACSAria™ III, BD Transduction Laboratories). Cells without expression (knockout) or reconstituted *TGFA* were individually separated in 96-well plates. TGFα expression in the different clones was analyzed by Western.

### Generation of monoclonal antibodies

The generation of anti-human TGFα monoclonal antibodies 5F1 and 16B10 was made under contract with the company BIOTEM (Apprieu, France). For the in-house production of the monoclonal antibodies, hybridomas provided by BIOTEM were cultured in 75 cm^2^ flasks in DMEM complete medium supplemented with 10% of FBS and 1% of non-essential amino acids (NEAA) (Thermo Fisher Scientific). After two days, cells were collected, centrifuged for 3 min at 1,200 rpm, and re-cultured in hybridoma-serum-free media (Thermo Fisher Scientific). After 3–4 days, hybridoma’s culture media were collected, clarified by low-speed centrifugation, and stored at -20ºC. Purification of the anti-TGFα monoclonal antibodies was performed by protein A-Sepharose^®^ chromatography. One liter of hybridoma culture media was run across a 4 mL of protein A-Sepharose^®^ column overnight at room temperature. After this time, all media was collected and consecutive washes of the column were performed with 10 mM Tris pH 8.0, 0.5 M NaCl, until the absorbance at 280 nm was less than 0.01. The antibodies were eluted from the column by incubation with 0.1 M glycine, pH 2.7 and collected fractions were neutralized with 150 µL of 1 M Tris-HCl pH 8.0. The absorbance of each fraction was then measured. Fractions with absorbance greater than 0.2 at 280 nm were pooled and concentrated by ultrafiltration with a 100 K Amicon^®^ Ultra-4 filter (Milipore).

### Preparation of DM-1, DXd and MMAF-coupled ADCs

For the preparation of 5F1-DM1 and 16B10-DM1 ADCs, the Safe & Easy Toxin (SET™) ST0101 SMCC-DM1 reagent was used (Levena Biopharma, San Diego, CA, USA) [29, 30]. For the preparation of 5F1 and 16B10 coupled to DXd or MMAF, the antibody buffer was exchanged to PBS, pH 7.4, using a PD-10 desalting column (GE Healthcare). The antibody was then heated to 37ºC for 10 min in a thermoblock, and 8 molar equivalents of freshly prepared tris(2-carboxyethyl) phosphine (TCEP) were added to the antibody solution. This reaction mixture was incubated for 2 h at 37ºC. Then, 20 molar equivalents of payloads (either Vc-MMAF or Maleimide-GGFG-DXd) were added, and the conjugation reaction was incubated at room temperature for 1 h. A PD-10 column was used to remove any free low molecular weight reagent, and the different ADCs were sterilized using a 0.22 μm low retention protein filter (Merck-Millipore). The final concentration of each conjugated antibody was measured using the NanoDrop™ spectrophotometer. Coupling of the payloads to the mAbs was analyzed by Western blotting using anti-payload antibodies. The integrity of the heavy and light chains of the antibodies was assessed using stain-free gels and a ChemiDoc apparatus (Bio-Rad Laboratories, Hercules, CA, USA).

### Protein extraction, Immunoprecipitation and Western blot

The procedures for preparing cell extracts, protein immunoprecipitation and Western blotting have been described [26, 29]. Briefly, cultured cells were washed with PBS and lysed in an ice-cold lysis buffer (20 mM Tris-HCl pH 7.0, 140 mM NaCl, 50 mM EDTA, 10% glycerol, 1% Nonidet P-40, 1 µM pepstatin, 1 µg/ml aprotinin, 1 µg/ml leupeptin, 25 mM β-glycerol phosphate, 50 mM sodium fluoride, 1 mM phenylmethylsulfonyl fluoride, 1 mM sodium orthovanadate). Lysates were centrifuged at 10,000 x*g* at 4ºC for 10 min. Supernatants were then quantified and transferred into new tubes with the corresponding antibody and protein A-Sepharose^®^ or GammaBind G-Sepharose^®^ for at least 2 h at 4ºC. Immunocomplexes were centrifuged at 10,000 x*g* for 15 s, followed by three washes with 1 mL of cold lysis buffer. Samples were run on SDS-PAGE gel, and the separated proteins were transferred to polyvinylidene difluoride (PVDF) membranes (Millipore Corporation, Bedford, MA, USA). Membranes were then blocked for at least 1 h in TBST (Tris [pH 7.5] 100 mM, NaCl 150 mM, 0.05% Tween 20) with 1% of BSA and then incubated with the desired antibody for at least 1 h. After this time, membranes were washed three times with TBST, 7 min each, and incubated with the HRP-conjugated anti-mouse or anti-rabbit secondary antibody for 30 min. After washing three times with TBST, blots were visualized by autoradiography or digital capture using a Chemidoc apparatus (Bio-Rad Laboratories, Hercules, CA, USA) [31]. Stain-free gels were performed by adding 50 µl of 2,2,2-Trichloroethanol to 10 ml of the SDS-PAGE gel solution and proteins visualized using a ChemiDoc apparatus after electrophoresis.

### Cell surface Immunoprecipitation

To immunoprecipitate cell surface TGFα, cells were grown on 100 mm plates, washed twice with Krebs-Ringer-HEPES buffer (KRH: 150 mM NaCl, 5 mM KCl, 2 mM CaCl_2_, 5 mM MgSO_4_, 1.2 mM KH_2_PO_4_; 6 mM glucose; 50 mM HEPES, pH 7.4) and incubated for 2 h at 4ºC in 2 mL of KRH with 1.5 µg/mL of the anti-human TGFα antibodies (5F1 or 16B10). This step was followed by two washes with cold PBS, and cells were then lysed and clarified following the standard protocol. Once the total protein of each sample was quantified, cell extracts were immunoprecipitated with protein A-Sepharose^®^ or GammaBind G-Sepharose^®^ for 30 min at 4ºC. After this time, the immunocomplexes were washed and loaded into SDS-PAGE gels.

### Detection of soluble TGFα in culture media

Ten-twelve 100 mm plates of each cell line were cultured in a complete medium until confluence. Twenty-four hours before the experiment, the culture medium was changed to DMEM without FBS. Media were collected, centrifuged at 1,200 rpm for 3 min to remove cell debris, and concentrated by ultrafiltration with a 3 K Amicon^®^ Ultra filter (Merk Millipore) to a final volume of 150 µL. SDS-PAGE loading buffer was added to the conditioned media and the samples were run in a 10–15% gradient polyacrylamide gel. TGFα in the different concentrated media of each cell line was detected by Western blotting using the R163 polyclonal anti-mature TGFα antiserum.

### Immunofluorescence microscopy

Cells were plated on glass coverslips inserted into 35 mm dishes and treated with 10 nM of anti-TGFα antibodies for 12 h. For colocalization experiments, chloroquine (10 mM, Sigma-Aldrich) was added 3 h before the antibody. After this time, cells were washed with PBS/Ca2+/Mg2+ (PBS/CM: 1 mM CaCl_2_, 0.5 mM MgCl_2_ in PBS) and fixed in 2% *p*-formaldehyde for 30 min at room temperature. Later, cells were washed with PBS/CM, quenched with 50 mM NH_4_Cl, permeabilized (0.1% Triton-X100, 0.2% BSA), and then incubated for 1 h in blocking solution (PBS/CM with 0.2% BSA). Coverslips were then incubated with the anti-LAMP1 (1:200 dilution) antibody in a blocking solution for 1 h at room temperature. After three washes for 7 min each in blocking solution, the coverslips were incubated with Cy3-cojugated anti-mouse and/or Cy2-conjugated anti-rabbit antibodies for 30 min. After this incubation, coverslips were washed three times for 5 min each, in PBS with 0.2% BSA, stained with DAPI, and mounted. Samples were analyzed by confocal immunofluorescence microscopy using a Leica TCS SP5 System (Leica Microsystem CMS, Wetzlar, Germany).

### Xenograft studies

Animal handling was performed in a pathogen-free area at the institute’s animal facility, following legal and institutional guidelines in accordance with European (Directive 2010/63/EU) and Spanish (Law 6/2013) legislation regarding the protection of animals used for scientific purposes. Female BALB/cOlaHsd-Foxn1-nu mice of 6 weeks old were obtained from Envigo Laboratories (Barcelona, Spain). A total of 5 × 10⁶ NP29 cells, suspended in 50 µL of DMEM supplemented with 10% fetal bovine serum and 50 µL of Matrigel (BD Biosciences), were subcutaneously injected into the animal’s flank. When tumors reached 150–200 mm^3^, mice were randomized into 3 groups (*n* = 6) and treated intraperitoneally once a week with vehicle, 16B10 and 16B10-MMAF (3.33 mg/kg). Tumor diameters were serially measured with a digital caliper (Proinsa, Vitoria, Spain), and body weight was measured twice a week. Tumor volume was calculated using the following formula: V = (L x W^2^)/2, where V = volume (cubic milimeters), L = Length (milimeters), and W = width (milimeters). Animals were sacrificed on day 21 of treatment by CO_2_ inhalation.

Tumor tissues used to generate the PDX were collected from patients who had undergone surgical resection for PDAC at the Bellvitge Hospital, Barcelona, Spain and the study was approved by the Ethical Committee of University Hospital of Bellvitge CEIC 02/04 and written informed consent was obtained from all patients for the use of their tissues. To generate PDX, 2 mm^3^ fragments of human pancreatic ductal adenocarcinomas were implanted in the tail of the pancreas of nude mice under anesthesia with isoflurane. Tumor formation was monitored by palpation. Successive passages were performed until the fifth passage, when the tumor was considered perpetuated. Mice were sacrificed when tumors measured approximately 1 cm of diameter.

### In Silico studies

Expression of TGFα in tumors and normal tissues was evaluated using the online platforms TNMplot, GEPIA2, Firebrowse and UCSC Xena. Data presented in the paper were downloaded from the web pages in November 2024. The expression analyses of TGFα in cancer cell lines were conducted using the public genomic data available in the Cancer Cell Line Encyclopedia (CCLE).

### Statistical analysis

Statistical analyses were performed using the software GraphPad Prism 8 (San Diego, Ca, USA). Normality distribution and homogeneity of variances were checked by the Shapiro-Wilk and Levene tests, respectively. The Mann Whitney U-test was used when continuous variables between two groups wanted to be compared. Differences were considered statistically significant when *p* < 0.05.

## Results

### Expression of TGFα in pancreatic tumors and cell lines

**Fig. 1:**
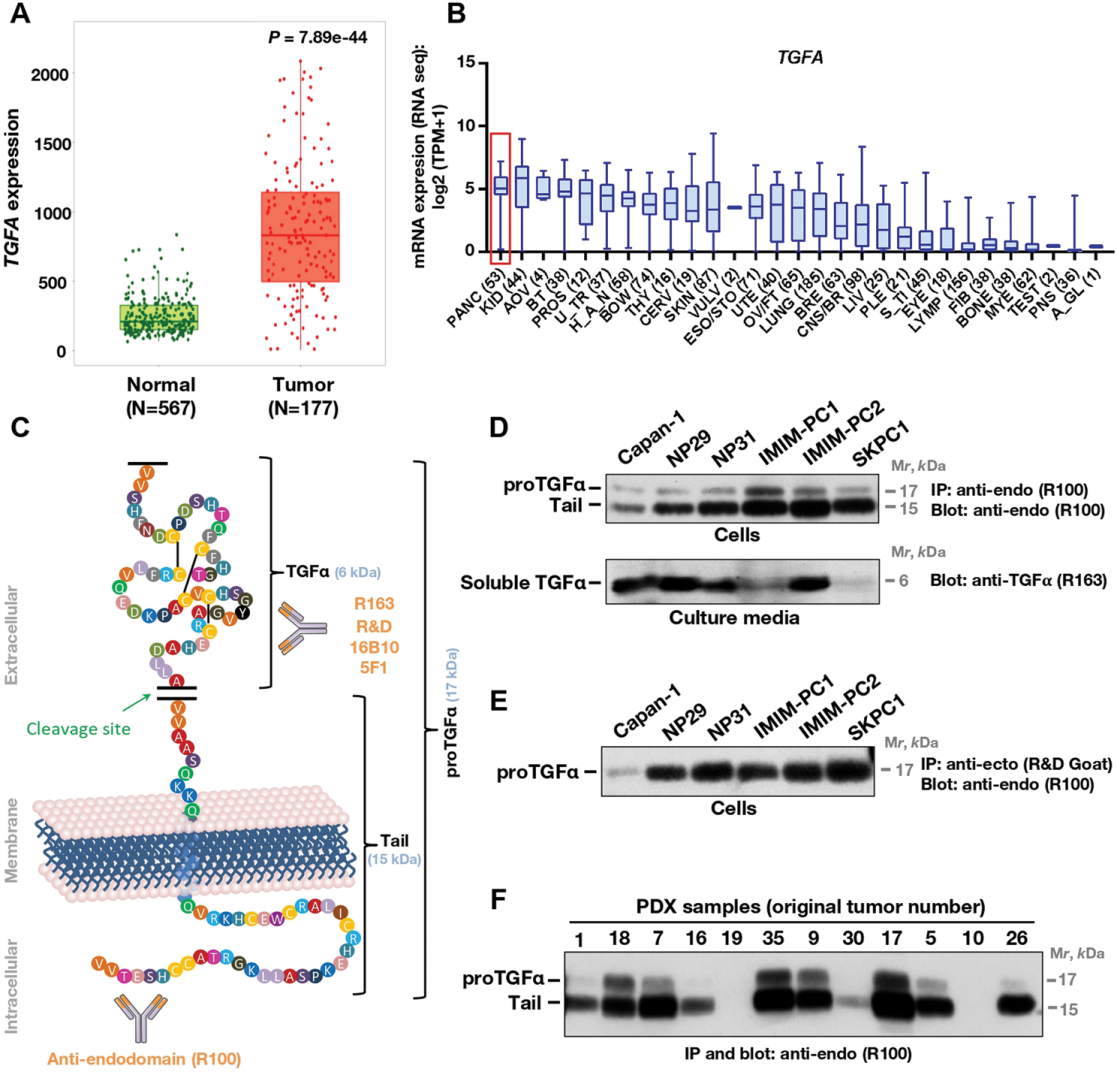
*TGFΑ* expression in pancreatic cancer. (**A**) Box plot showing *TGFA* gene expression levels in normal and tumor tissue from pancreatic cancer patients. Data was obtained from the TNMplot database. (**B**) Expression of *TGFA* in human tumor-derived cell lines, obtained from RNA-Seq data available in the Cancer Cell Line Encyclopedia (accessed february 2023). The number of cell lines analyzed in each tissue is indicated in parentheses. A red box indicates the expression of the gene in pancreatic cancer cells. H_A_N: head and neck, U_TR: urinary tract, PLE: pleura, BT: biliary tract, PROS: prostate, ESO/STO: esophagus/stomach, KID: kidney, CERV: cervix, PANC: pancreas, AOV: ampulla of vater, THY: thyroid, LIV: liver, FIB: fibroblast, UTE: uterus, OV/FT: ovary/fallopian tube, CNS/BR: central nervous system/brain, BOW: bowel, S_TI: soft tissue, BRE: breast, PNS: peripheral nervous system, VULV: vulva, TEST: testis, A_GL: adrenal gland, MYE: myeloid, LYMP: lymphoid. (**C**) Schematic representation of proTGFα, including the different domains, its proteolytic cleavage site, and the different antibodies used. (**D**) Western analyses of the expression of TGFα in pancreatic cancer cell lines. One mg of total extract of each cell line was immunoprecipitated with the anti-endodomain polyclonal antibody (R100) followed by Western blot detection with the same antibody (top panel). For the detection of soluble TGFα, cells were allowed to condition their culture media (without serum) overnight, using ten-twelve 100 mm plates. The culture media were collected, centrifuged, and concentrated to finally analyze the total amount of TGFα secreted into the culture medium by Western using the anti-ecto domain polyclonal antibody R163. (**E**) Cell surface immunoprecipitation of TGFα. Cells were incubated for 2 h at 4ºC with the anti-ectodomain R&D polyclonal antibody, and lysed. One mg of cell extract was immunoprecipitated by addition protein A-Sepharose, and finally analyzed by Western blot using the anti-endo domain polyclonal antibody R100. (**F**) Expression of TGFα forms in patient-derived xenografts. After dissection, tumoral samples were homogenized in lysis buffer and 1 mg of protein used for immunoprecipitation with the R100 antibody, followed by Western with the same antibody

Analysis of *TGFA* gene expression using the TNMPlotter online tool indicated that *TGFA* was significantly (*P* = 7.89e-44) overexpressed in the tumoral tissues (*N* = 177) when compared to normal pancreatic tissue (*N* = 252) (Fig. 1A). That conclusion was also supported by comparing normal versus neoplastic pancreatic tissues using other online tools, which included GEPIA2, UCSC-Xena or Firebrowse (supplementary Figs. 1A-C). Moreover, pancreatic adenocarcinomas were among the tumoral tissues in which expression of *TGFA* was higher when compared to other neoplasias (supplementary Fig. 1A, B, D, and E). We also explored human tumoral cell lines for the expression of *TGFA* using the CCLE portal. In line with in silico data obtained from patient samples, pancreatic cancer cell lines expressed *TGFA* to levels above those expressed by most of the cell lines available in the database (Fig. 1B).

The expression and molecular forms of TGFα were analyzed in pancreatic cancer cell lines, as well as in patient-derived xenografts (PDXs). For that purpose, different antibodies that recognize epitopes located in the extracellular or intracellular domains of proTGFα were used (Fig. 1C and supplementary Fig. 2). Immunoprecipitation of lysates obtained from six different cell lines with the anti-endodomain antibody R100, followed by Western with the same antibody, showed the presence of a 17 kDa form, corresponding to transmembrane proTGFα (Fig. 1D, top Western), together with a 15 kDa form. The presence of the 15 kDa form was consistent with proteolytic cleavage of proTGFα, which should generate soluble TGFα [32]. To verify this, the culture media from the different cell lines was conditioned overnight in the absence of serum, and concentrated. These concentrated media were run on high percentage SDS-PAGE gradient gels, and TGFα detected by Western using an antibody (R163) raised in rabbits using mature human TGFα. These studies allowed detection of soluble TGFα in the culture supernatants of the cell lines studied, although the amounts were variable (Fig. 1D, bottom Western). Cell surface immunoprecipitation studies using a commercial (R&D Systems) goat polyclonal anti-ectodomain antibody showed that all the cell lines expressed the 17 kDa form (Fig. 1E). As expected, in these cell surface immunoprecipitations the 15 kDa band was not precipitated by the anti-ectodomain antibody.

Expression of TGFα forms was also analyzed in patient-derived xenografts (PDXs) obtained from twelve different pancreatic adenocarcinomas. Tissue extracts were immunoprecipitated with the anti-endodomain antibody R100, followed by Western analysis with the same antibody. These studies showed that ten out of the twelve tumors expressed TGFα, with a pattern consistent with larger amounts of the 15 kDa tail form with respect to 17 kDa proTGFα (Fig. 1F).

### Endogenous TGFα promotes proliferation of pancreatic cancer cells

**Fig. 2:**
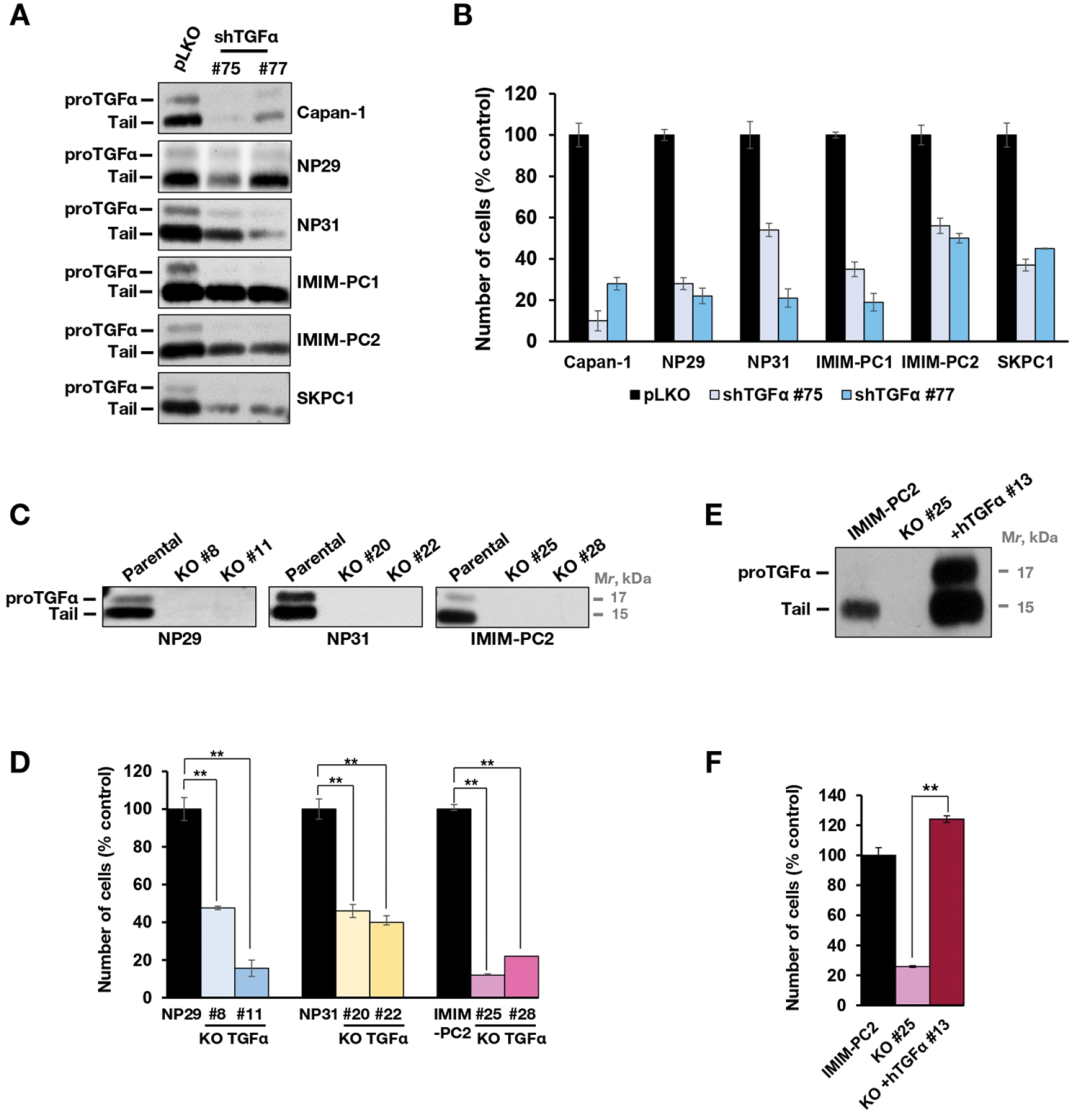
TGFα facilitates the proliferation of pancreatic cancer cell lines. (**A**) Cells were infected with viruses including two different short hairpin sequences against *TGFA* (sequences #75 and #77) and the control vector (pLKO). Cell extracts were immunoprecipitated with the anti-endodomain polyclonal antibody (R100) followed by Western blot with the same antibody. (**B**) Effect of TGFα knockdown on cell proliferation. Cells were infected with the different shRNA and counted 5 days after puromycin selection. Results are represented as the percentage of the mean ± SD of triplicates relative to the control shRNA (pLKO). The graph shows results from a representative experiment that was repeated twice. (**C**) Knockout of *TGFA* in different pancreatic cancer cell lines. Parental NP29, NP31 and IMIM-PC2 cells and two different knocked out clones for *TGFA* were lysed. One mg of cell extract was immunoprecipitated with the anti-endodomain antibody R100 and blots incubated with the same antibody. (**D**) Effect of *TGFA* knockout on cell proliferation. The different CRISPR/Cas9 clones together with their parental cell lines were seeded in 6-well plates and counted after 5 days. The results are represented as the percentage of the mean ± SD of triplicates. The assay is representative of three independent experiments. **P < 0.01, calculated by Mann-Whitney U test. (**E**) Reconstitution of *TGFA* gene expression in IMIM-PC2-KO#25. The CRISPR/Cas9 clone was transduced with a pLZR_hTGFα vector and selected by single-cell sorting. The clone with reconstituted *TGFA* (IMIM-PC2 + hTGFα #13) was lysed, TGFα was immunoprecipitated, and the total amount of the protein was compared with the KO and the parental cell line by Western blot. (**F**) The proliferative capacity of the cell line reconstituted with *TGFA* was assessed on day 5 by cell counting. The results are represented as the percentage of the mean ± SD of the triplicates with respect to the parental cell line. The assay is representative of three independent experiments. **P < 0.01, calculated by Mann-Whitney U test

To explore the role of endogenous TGFα in the proliferation of pancreatic cancer cells, loss of function studies were carried out. First, the expression of TGFα was down regulated by RNAi using a lentiviral system. Preliminary studies using five different shRNAs allowed the selection of the two sequences, numbers 75 and 77, that caused the best knockdown of proTGFα. Infection of the six pancreatic cell lines with lentiviral particles including the two knockdown sequences resulted in downregulation of proTGFα expression (Fig. 2A). The effect of proTGFα knockdown on the proliferation of the pancreatic cancer cell lines was assessed by cell counting experiments. These studies showed that proTGFα knockdown provoked a substantial decrease in the proliferation of those cells (Fig. 2B).

To corroborate that TGFα was a relevant factor in the regulation of pancreatic cancer cell proliferation, we eliminated its expression by CRISPR/Cas9 in three of the pancreatic cancer cell lines (Fig. 2C), and two clones of each cell line were selected to assess the effect of TGFα loss on cell proliferation. As shown in Fig. 2D, the deletion of *TGFA* resulted in a significant inhibitory action on the proliferation of the cells. These data confirmed that TGFα expression facilitated the proliferation of pancreatic cancer cell lines. Moreover, reconstitution of TGFα expression in one of the clones in which *TGFA* had been formerly deleted (IMIM-PC2#25, clone 13, Fig. 2E), rescued proliferation (Fig. 2F). It is worth commenting that in the rescued clone, proTGFα levels were higher than in parental IMIM-PC2 cells (Fig. 2E).

### Generation of monoclonal antibodies recognizing TGFα

The fact that proTGFα is overexpressed in pancreatic cancer and participated in the proliferation of pancreatic cancer cell lines, opened the possibility of using it as a therapeutic target. Moreover, the fact that proTGFα is a membrane protein, raised the possibility of using it as a target of antibody-drug conjugates. Considering all of the above. a strategy based on the use of antibodies was considered to directly and specifically target TGFα. Several commercial monoclonal antibodies against mature human TGFα were tested, but none of them resulted satisfactory in terms of capability to recognize native TGFα. Because of that, monoclonal antibodies directed to human TGFα were raised. A schematic description of the steps used to raise those antibodies is shown in supplementary Fig. 3. Seven monoclonals were able to recognize human native TGFα in immunoprecipitation experiments (supplementary Fig. 3), and from them, two monoclonals (5F1 and 16B10) were selected for further studies.

**Fig. 3:**
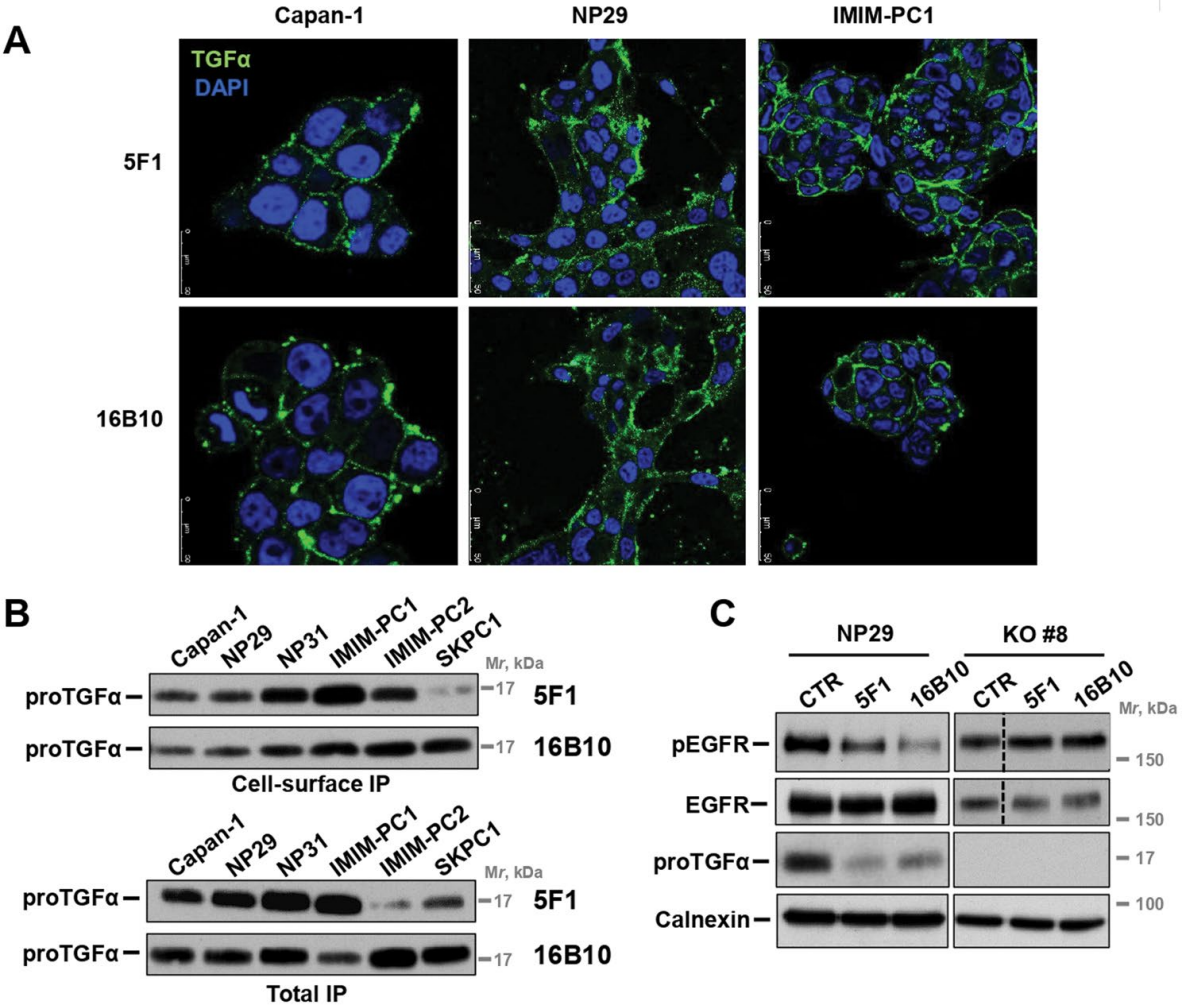
TGFα the monoclonal antibodies recognize cell surface proTGFα. (**A**) Representative PDAC cell lines (Capan-1, NP29 and IMIM-PC2) were seeded on coverslips and treated for 30 min on ice with 2.5 nM of 5F1 or 16B10 mAbs. After washing, cell-bound antibodies were visualized by immunofluorescence microscopy using and Alexa Fluor 488-conjugated anti-mouse antibody. Scale bar: 50 µM. (**B**) Cell surface (top) and total (bottom) immunoprecipitation of TGFα by the monoclonal antibodies. For cell surface, immunoprecipitation, cells were treated with 2.5 nM of either 16B10 or 5F1 for 2 h at 4ºC, washed with PBS and then lysed. Cell extracts were precipitated with protein A-Sepharose and analyzed by Western blot with the R100 anti-endodomain antibody. For total IP, 1 mg of cell extract was also immunoprecipitated with 3 µg of each mAb and then analyzed by Western with the R100 antibody. (**C**) Effect of 5F1 or 16B10 on total and active levels of EGFR, and the levels of TGFα. NP29 and the CRISPR/Cas9 clone #8 were treated with 2.5 nM of each mAb for 5 days. Cells were lysed, and EGFR or TGFα immunoprecipitated. The phosphorylation status of EGFR was detected using the anti-pTyr99 antibody. Calnexin was used as a loading control

To analyze whether 5F1 and 16B10 antibodies recognized proTGFα in vivo, cell surface immunofluorescence staining as well as cell surface immunoprecipitation experiments were performed. In the immunofluorescence experiments, after incubation of cell monolayers with 5F1 or 16B10 antibodies, followed by washing and fixation, a staining pattern compatible with surface-bound antibodies was observed (Fig. 3A). In cell surface immunoprecipitation experiments, cells were incubated with the antibodies for two hours, washed and lysed. The lysates were precipitated by incubation with protein A-Sepharose to pull down the antibodies and associated proTGFα. As shown in Fig. 3B, both antibodies were able to pull down the 17 kDa form, indicating that the antibodies were able to interact with membrane-bound proTGFα. Moreover, in a conventional immunoprecipitation experiment, the antibodies also immunoprecipitated proTGFα after cell lysis (Fig. 3B, total IP). In these two different conditions (cell surface or total cell lysate immunoprecipitation), 5F1 and 16B10 precipitated the 17 kDa form, but were unable to precipitate the 15 kDa form, devoid of the TGFα region. Together, these results indicated that the two monoclonal antibodies recognized native TGFα even when included within its precursor 17 kDa proTGFα transmembrane form.

Both antibodies were able to reduce the amount of pEGFR in NP29 parental cells, but did not affect pEGFR levels in a proTGFα NP29-derived knockout clone (#8). Interestingly, treatment with 5F1 or 16B10 antibodies reduced the amount of total proTGFα (Fig. 3C). This latter result was relevant as it suggested that the monoclonal antibodies could provoke down regulation of proTGFα, likely by increasing its internalization and degradation. That fact reinforced the possibility of using proTGFα as an ADC target.

### Construction and efficacy of ADCs targeting ProTGFα

**Fig. 4:**
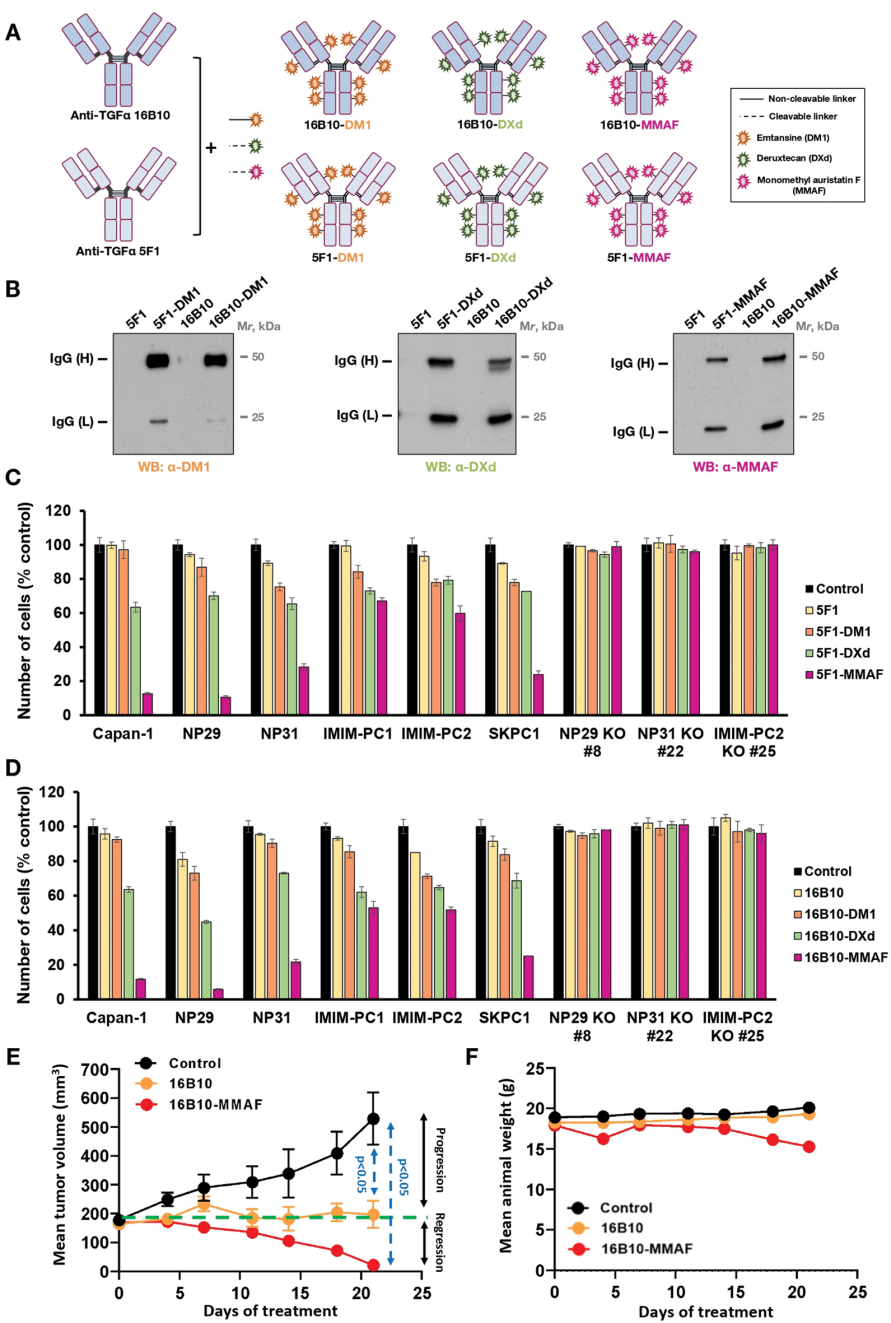
Preparation and cytotoxic activity of different ADCs against TGFα. (**A**) Schematic representation of the different ADCs generated against TGFα. (**B**) Western blot analyses for assessment of the conjugation of the cytotoxic payloads to the anti-TGFα mAbs. Forty nanograms of each ADC as well as the unconjugated mAbs were loaded in 12% SDS-PAGE gels. The cytotoxic payload coupled to the light (IgG, L) and heavy (IgG, H) chains of the ADCs were analyzed by Western using anti-DM1, anti-DXd or anti-MMAF antibodies. C, D. Cells were seeded in 6-well plates and treated with 2.5 nM of the naked antibodies and the different ADCs. After 5 days, the effect of the 5F1 (**C**) or 16B10 (**D**) ADCs on proliferation was assessed by cell counting. The results are represented as the percentage of the mean ± SD of the triplicates with respect to the untreated controls. The result is representative of three independent experiments. (**E**). In vivo effectiveness of anti-TGFα and anti-TGFα-MMAF on NP29 xenografts. NP29 cells were injected in Balb/c nude mice and treated once a week with 3.3 mg/kg of 16B10 or 16B10-MMAF antibodies for 21 days. The tumor volume of each group (*n* = 6) was measured twice a week. Results are represented as the mean ± SEM and the *P*-value was calculated using the Mann-Whitney U-test. (**F**). Effect of the 16B10 and 16B10-MMAF treatment on the body weight of mice. Data are presented as the mean ± SD for each group of mice

To explore the possibility of using proTGFα as an ADC target, 5F1 and 16B10 monoclonal antibodies were conjugated to three payloads used in the clinic: emtansine (DM1), deruxtecan (DXd) or monomethylauristatin-F (MMAF)(Fig. 4A). To parallel the clinical situation, DM1 was coupled to the antibodies through an uncleavable linker, while in the case of DXd and MMAF a cleavable linker was used. Electrophoretic analyses showed that coupling of the antibodies to the payloads caused a small retardation in the migration of the resulting ADCs, indicative of an increase in molecular weight of the ADCs with respect to the uncoupled antibodies (supplementary Fig. 4A, Stain-free gels). To verify that the payloads were coupled to the antibodies, Western blotting analyses using antibodies raised against DM1, DXd, and a commercial one that recognized MMAF were performed. These studies demonstrated that the different payloads were covalently bound to 5F1 or 16B10 (Fig. 4B and supplementary Fig. 4A and B). In contrast, no signals were obtained in the case of the uncoupled nude forms of these monoclonal antibodies. DM1 coupled better to 5F1 and 16B10 heavy chains than to the light chains, and the amount of coupling was similar to that of commercially available trastuzumab-DM1, although in the latter the payload coupled similarly to both antibody chains (supplementary Fig. 4B).

Cell counting studies were performed to assess the antiproliferative activity of the different ADCs. In these studies, we also included the nude 5F1 or 16B10 antibodies for comparison, in addition to the six different ADCs, three derived from each mAb. As shown in Figs. 4C and D, the antiproliferative effect of the ADCs was more pronounced than that of the nude antibodies in all six wild type cell lines. Among the different ADCs tested, the antibodies coupled to MMAF resulted the most potent and efficient, followed by those coupled to DXd. That the action of these antibodies was dependent on proTGFα expression was demonstrated by the fact that neither the nude 5F1 or 16B10 antibodies nor the ADCs derived from them had any significant effect on cells lacking proTGFα (NP29-KO#8, NP31-KO#22 and IMIM-PC2-KO#25). Moreover, reconstitution of proTGFα expression in IMIM-PC2-KO#25 cells restored the sensitivity to 16B10-MMAF (supplementary Fig. 4C).

Given the fact that MMAF-coupled antibodies resulted the most potent and efficient in vitro, we selected them for in vivo evaluation of their potential antitumoral action. The antitumoral effect of 16B10 as well as 16B10-MMAF was evaluated in nude mice injected with NP29 cells. Treatment of mice with 16B10 caused stabilization of tumor size, and the tumors retained a volume which did not vary along the duration of the experiment (Fig. 4E). In mice treated with the ADC, the tumors reduced in size with respect to measurements made at the beginning of the treatment, and were not detected at the end of the experiment (Fig. 4E). The weight of the mice remained stable along the duration of the experiment with only a minor decrease in animals treated with 16B10-MMAF (Fig. 4F). These results indicated that the effect of 16B10-MMAF in mice was more pronounced than in the case of the nude antibody, in line with the results obtained in vitro.

**Fig. 5:**
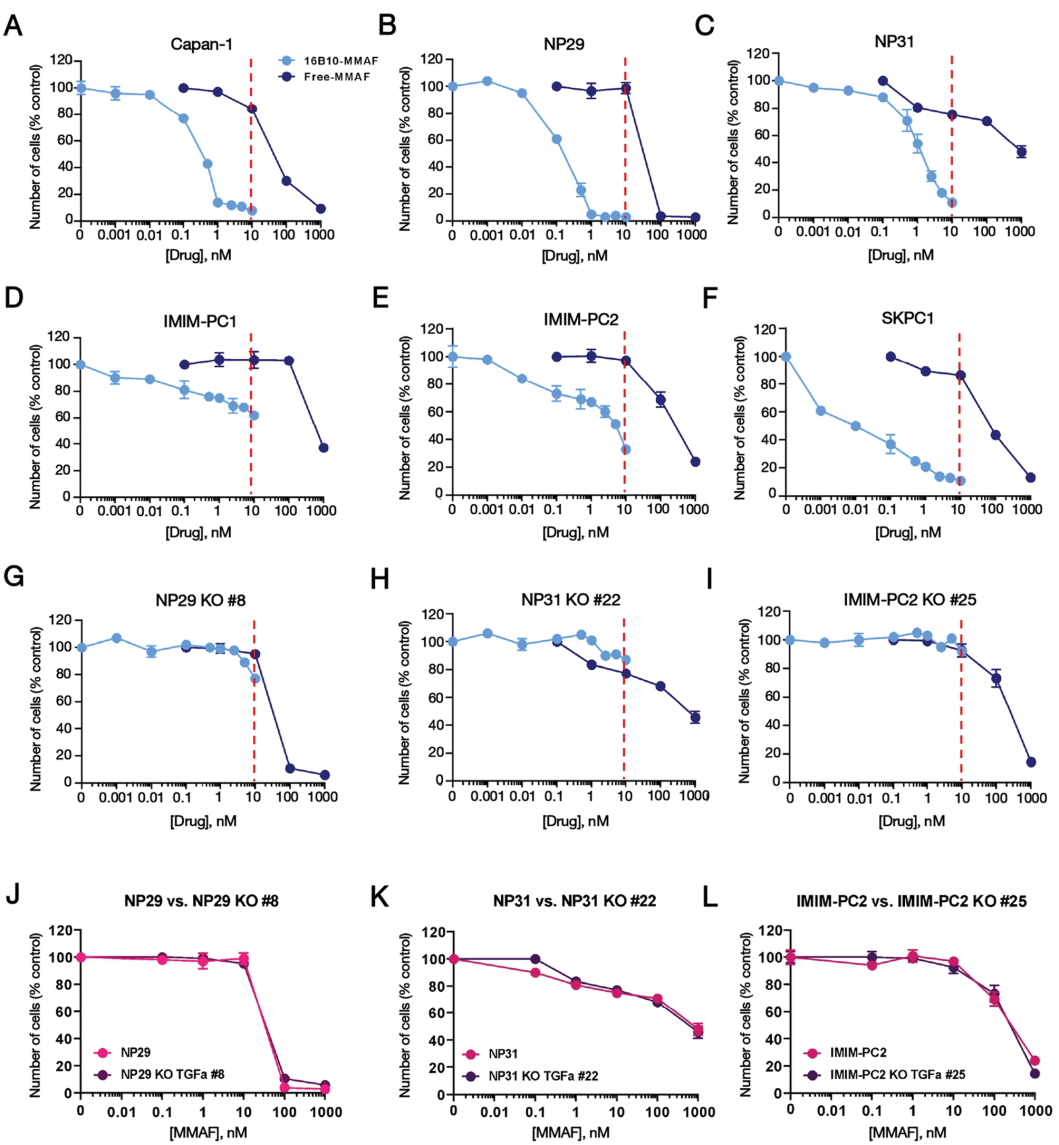
Dose-response analysis of the antiproliferative effect of 16B10-MMAF and free MMAE in pancreatic cancer cell lines. **A**-**I**. Cell lines were treated with the indicated concentrations of the ADC or the cytotoxic payload for 5 days. The effect on cell proliferation was assessed by cell counting, and the results were represented as the percentage of the mean ± SD of triplicates of an experiment repeated three times. Red dashed lines mark the concentration of 10 nM. **J**, **K**, **L**. Comparative effect on cell proliferation of the cytotoxic payload MMAE in CRISPR/Cas9 clones compared to its parental cell line. Data was represented as mean ± s.d. of triplicates of an experiment repeated three times

The antiproliferative action of MMAF compared to 16B10-MMAF was then evaluated. As shown in Fig. 5, the payload exerted an inhibitory effect on all the cell lines studied. However, the potency of 16B10-MMAF was substantially higher than that of the free payload (Fig. 5A-I), reaching IC_50_ values below 10 nM (supplementary Fig. 5). In contrast, the IC_50_ values of the free payload were always above 10 nM. These dose-response analyses confirmed the resistance of TGFα-knocked out cells to the action of 16B10-MMAF (Fig. 5G-I). Of note, the sensitivity of the knocked-out cell lines to the free payload was similar to that of the wild type cell lines (Fig. 5J-L).

### Mechanism of action of MMAF-coupled anti-proTGFα ADC

**Fig. 6:**
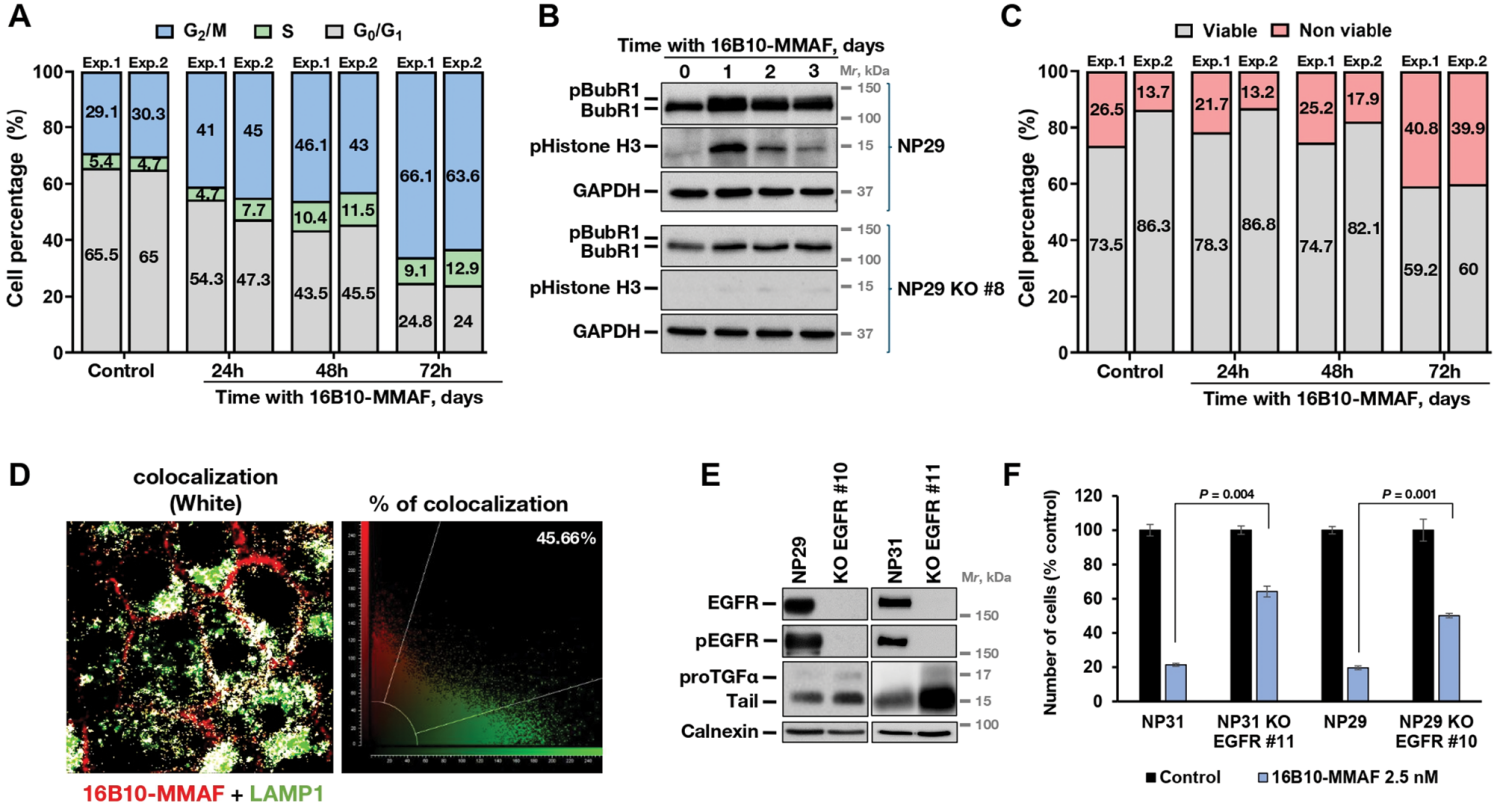
Mechanism of action of 16B10-MMAF. (**A**) Effect of 16B10-MMAF on the cell cycle. NP29 cells were treated with 16B10-MMAF (10 nM) for the indicated times, stained with PI, and analyzed by FACS. Histograms represent the percentage of cells in each cell cycle phase from two independent experiments. (**B**) Effect of 16B10-MMAF on mitotic marker proteins. The cell lines were seeded in 100 mm plates and treated with 2.5 nM of 16B10-MMAF ADC. Cells were lysed at the indicated times, and BUBR1 or pHistone H3 assessed using the corresponding antibodies. GAPDH was used as a loading control. (**C**) Effect of 16B10-MMAF on cell death. NP29 cells were treated with 16B10-MMAF (10 nM) for the indicated times, and apoptosis was evaluated by Annexin V-FITC/PI double staining followed by flow cytometry analysis. Histograms illustrate the proportions of viable (Annexin V negative/PI negative) and non-viable cells from two independent experiments. (**D**) Colocalization analysis of 16B10-MMAF and LAMP-1 in NP29 cell line. Cells were treated with 2.5 nM of 16B10-MMAF (red) for 12 h at 37ºC and after fixation and permeabilization, incubated with an anti-LAMP-1 antibody (green). The points of colocalization between LAMP-1 and the ADC are shown in white (left). Colocalization analysis was done with Leica Application Suite Advanced Fluorescence, which generated the scatter plots of acquired images (right). (**E**) EGFR and TGFα expression levels in different *EGFR* CRISPR/Cas9 clones. One mg of NP29 EGFR KO #10, NP31 EGFR KO #11 and their corresponding wild type cell lines was immunoprecipitated with an anti-EGFR antibody. Phosphorylated EGFR was detected by Western using the anti-p-Tyr99 antibody. Another mg of protein of these cell lines was also immunoprecipitated with an anti-TGFα antibody (R100) and analyzed by Western blot. Calnexin was used as loading control. (**F**) Effect of 16B10-MMAF in EGFR CRISPR/Cas9 or parental cell lines. NP29 KO EGFR #10, NP31 KO EGFR #11 and their corresponding parental cell lines were seeded in 6 well-plates and treated with 2.5 nM of the anti-TGFα ADC. Cells were counted 5 days after initiation of the treatment. The results are represented as the percentage of the mean ± SD of the triplicates of an experiment that was repeated two times. **P < 0.01, calculated by Mann-Whitney U test

The inhibition of cell proliferation by the proTGFα-directed ADCs could result from cell cycle arrest, increased apoptosis, or a combination of both. Popidium iodide staining was performed and the cell cycle profiles of cells treated with 16B10-MMAF compared to those of untreated NP29 cells. Treatment with 16B10-MMAF led to an increase in the proportion of cells in the G2/M phase (Fig. 6A). Biochemically, 16B10-MMAF treatment caused the accumulation of mitotic markers pHistone H3 and pBubR1 (Fig. 6B). These biochemical changes were not observed in NP29 KO#8 cells (Fig. 6B), which lack proTGFα expression. To investigate the potential induction of cell death by 16B10-MMAF, NP29 cells were treated with the ADC and double Annexin V-FITC/propidium iodide staining analyzed. The number of viable cells in the treated samples decreased after 72 h of treatment with the ADC (Fig. 6C).

The mechanism of action of ADCs includes internalization and intracellular delivery of the payload, which mainly occurs in the lysosomes. To explore whether 16B10-MMAF internalized and was directed to lysosomes, colocalization studies were carried out. Capan-1 cells were treated with 16B10-MMAF for 24 h and immunofluorescence studies performed to explore its colocalization with the lysosomal marker LAMP1. As shown in Fig. 6D, almost half of the ADC colocalized with that lysosomal marker. Similar results were obtained when using 5F1-MMAF (supplementary Fig. 6). Moreover, incubation with the lysosomotropic agent chloroquine, that increases lysosomal pH, favored the colocalization of 16B10-MMAF and LAMP1 (supplementary Fig. 6). These results indicated that the ADCs internalized and trafficked to the lysosomal compartment, where they are likely processed by the acidic proteases present in that compartment.

Since TGFα acts as one of the ligands of the EGFR, and the pancreatic cancer cell lines expressed the receptor (supplementary Fig. 7A), we reasoned that coexpression of the EGFR could facilitate the antiproliferative action of the anti-TGFα ADCs. In this respect, in silico analyses showed a correlation between *TGFA* and *EGFR* expression levels (supplementary Fig. 7B). To explore the possibility that EGFR expression may facilitate the antiproliferative action of 16B10-MMAF, we created cell lines derived from NP29 and NP31, devoid of the EGFR (Fig. 6E), and analyzed the effect of 16B10-MMAF on the parental and EGFR knockout cells. Cell proliferation studies showed that the antiproliferative action of 16B10-MMAF was impaired in the clones lacking the EGFR (Fig. 6F). These studies suggested that the antiproliferative action of the anti- TGFα ADCs could be facilitated by the presence of EGFR.

## Discussion

Transforming growth factor-alpha has long been recognized as a factor that may contribute to cancer development, particularly due to its role in EGFR activation [5]. This study expands on the understanding of TGFα in pancreatic cancer by analyzing its expression and function in both patient-derived tumors and cell lines. In the latter, loss-of-function studies using RNAi and CRISPR/Cas9 technology demonstrated that the reduction or elimination of *TGFA* significantly inhibited the proliferation of the six pancreatic cancer cell lines tested. Specifically, in the CRISPR/Cas9 knockout experiments, the ablation of *TGFA* led to a profound reduction in cell proliferation, underlining the contribution of TGFα to the growth of these cells. Moreover, reintroduction of TGFα into *TGFA*-knockout cells restored their proliferative potential, further corroborating the centrality of this growth factor in pancreatic cancer progression. The ability to rescue proliferation by reintroducing TGFα also highlights its potential as a therapeutic target, where inhibiting TGFα function could provide a means to stymie pancreatic tumor growth. Furthermore, the membrane-bound properties of proTGFα, together with its internalization capability, could be exploited to direct ADCs to proTGFα-expressing tumors, as demonstrated in this work.

Using multiple in silico tools, it was confirmed that *TGFA* is significantly overexpressed in pancreatic adenocarcinomas compared to normal pancreatic tissues. The observed overexpression suggests a potential role of TGFα in pancreatic tumor progression. This conclusion is also supported by genetic studies in mice which demonstrated that overexpression of TGFα may provoke the appearance of tumors in several tissues, including the pancreas [12–14]. The presence of TGFα in pancreatic cancer was not limited to its overexpression. In fact, our study also identified multiple molecular forms of TGFα, including the 17 kDa transmembrane form (proTGFα) and a 15 kDa tail fragment. In addition, a 6 kDa soluble form of TGFα, secreted by pancreatic cancer cell lines, was also detected in the culture media.

The membrane-bound nature of the TGFα precursor opened the possibility of leveraging this molecular characteristic with therapeutic purposes, i.e. by using proTGFα as an ADC target. To explore such possibility, the monoclonal antibodies 5F1 and 16B10 were conjugated to cytotoxic drugs commonly used in clinical ADCs [25]. The ADCs, particularly the ones that contained MMAF, demonstrated a significant antiproliferative effect on TGFα-expressing pancreatic cancer cells, with IC_50_ values significantly lower than those of the free drug or the unconjugated antibodies. These studies demonstrated that MMAF-loaded ADCs were highly effective in delivering their cytotoxic payload to TGFα-expressing cells, making them promising candidates for further development. The specificity of the ADCs for TGFα-expressing cells was confirmed by their lack of activity in *TGFA*-knockout cells. Moreover, reconstitution of TGFα expression in knockout cells restored sensitivity to the ADCs, further reinforcing the idea that TGFα is a viable target for ADC-based therapies. It is relevant to indicate that all the pancreatic cancer cell lines used in the present study carry *KRAS* codon 12 mutations. The effectiveness of the ADC in this scenario opens the possibility of using the ADC in other tumors expressing oncogenic *KRAS* mutations. In fact, our preliminary data has shown effectiveness of the ADC in lung cancer cell lines with *KRAS* mutations and that express proTGFα.

In vivo studies showed that the unconjugated antibody 16B10 stabilized tumor growth, but the ADC exerted a higher antitumoral effect, inducing tumor regression. Importantly, the treatment with 16B10-MMAF was well tolerated, with minimal weight loss observed in the treated animals. However, one of the limitations of the present study is the fact that the monoclonal antibodies raised against human TGFα failed to recognize the murine form, preventing the accurate evaluation of the on-target potential toxicities derived from the administration of the mAbs or ADCs. While this represents a limitation of our study, it is worth noting that mice deficient for *TGFA* develop to term, and have minor abnormalities in skin appendages [33, 34]. The most severe reported effect was corneal inflammation. Considering this phenotype, it is expected that the on-target toxicity would be tolerable in humans.

The mechanism by which the MMAF-coupled ADCs exerted their antiproliferative effects included internalization and lysosomal targeting. In the latter cellular compartment, the action of acidic proteases is expected to release the cytotoxic payload, ultimately leading to cell death [22, 35]. Cell cycle analysis revealed that treatment with 16B10-MMAF induced G2/M phase arrest, accompanied by the accumulation of mitotic markers such as pHistone H3 and pBubR1, which are indicative of mitotic arrest [36, 37]. This mitotic disruption was absent in cells lacking TGFα, underscoring the specific mechanism of action of the ADC.

The interaction of TGFα with its cellular receptor may be leveraged to increase the antiproliferative action of the anti-TGFα ADCs. In fact, EGFR expression facilitated the antiproliferative action of the anti-TGFα ADCs. It is possible that soluble TGFα may interact with the EGFR, whose internalization efficiency is high when compared to other ErbB family receptors, and TGFα-EGFR interaction may facilitate the uptake of the anti-TGFα ADCs. This possibility is indicated by the fact that EGFR knockout cells exhibited decreased sensitivity to the ADC treatment. However, in this work we did not explore such possibility in detail, and further work is needed in order to define to which extent part of the antiproliferative action of anti-TGFα ADCs may depend on the TGFα-ADC uptake mediated by the EGFR. Another possibility that may be considered is the fact that TGFα is expected to promote proliferative signaling by the EGFR, and such signaling may be compromised in cells treated with anti-TGFα antibodies.

In addition to the relevance of the studies herewith presented as a potential novel strategy for the therapy of pancreatic cancer, several other more generic concepts can be extracted from the present study. First, it demonstrates the suitability of membrane-anchored growth factors as ADC targets. While these factors may be proteolytically cleaved to release soluble mature polypeptides, the membrane-bound characteristics of their precursors may be exploited to facilitate targeting of the ADC to tumors in which the transmembrane factor is biosynthesized. Second, the growth promoting characteristics of some of these growth factors could potentially be attenuated by the antibodies used against them. Finally, the coexpression of the receptor and the membrane-bound factor may increase the uptake of the ADCs directed to the growth factor, augmenting the antitumoral action of the ADC. From a more generic point of view, these characteristics also open the possibility of targeting membrane-anchored growth factors overexpressed in other neoplasias, expanding the therapeutic armamentarium against those diseases. In the case of the studies herewith reported, the in vitro and in vivo results demonstrate the potent antitumor activity of ADC that target TGFα, providing a strong rationale for further preclinical and clinical investigation of ADCs targeting proTGFα as a therapeutic strategy for pancreatic cancer.

## Conclusion

This study highlights the overexpression and functional significance of TGFα in pancreatic cancer, making it a promising therapeutic target. The development of monoclonal antibodies and their subsequent conjugation to cytotoxic agents to create ADCs offers a highly specific and potent strategy for inhibiting pancreatic cancer growth. Among the tested ADCs, those conjugated to MMAF were particularly effective, providing a powerful new approach to pancreatic cancer treatment. The successful in vitro and in vivo targeting of TGFα supports further exploration of this approach in clinical settings.

## Electronic supplementary material

Below is the link to the electronic supplementary material.

Supplementary Material 1: Fig. 1. A, B. In silico data (from GEPIA2 in A, and Firebrowse in B) on *TGFA* expression in various tumors, compared to normal tissue. The arrow indicates the pancreatic normal and tumoral tissues, which are also marked by a blue rectangle. In red, the tissues in which *TGFA* expression is significatively higher in the tumoral vs. normal samples. Abbreviations of the tissues detailed in the respective web pages. C. In silico data (from UCSC-Xena) on *TGFA* expression in pancreatic tumors (primary site and metastatic), compared to normal tissue. D. Ranking of *TGFA* expression in different tumors, as obtained from the UCSC-Xena online tool. A blue rectangle marks pancreatic tumors. E. Ranking of *TGFA* expression in different tumors, as obtained from the cBioPortal online tool. The arrow marks pancreatic tumors.

Supplementary Material 2: Fig. 2. Representative diagram of proTGFα domains, indicating the molecular weight corresponding to different forms, the amino acid sequence, as well as the different antibodies generated against this protein (antibody name, species, type, and source).

Supplementary Material 3: Fig. 3. Generation of the monoclonal antibodies. After the initial immunization (1), three of the injected mice raised antibodies able to recognize proTGFα when the culture supernatants were tested in immunoprecipitation experiments (2). One of these mice, #4, gave the best results (2) and was then selected to carry out fusions and selection of oligoclonal populations (3). ELISA of mature TGFα used as the screening strategy for the testing culture supernatants from 85 different oligoclonal populations (4), and then positive oligoclonal cultures were used to test them in immunoprecipitation experiments of native proTGFα (4) using extracts of NP29 cells. These studies led to the selection of eight of those populations, which upon a second round of expansion and single cell cloning (5) allowed expansion of four different subclones (6). After single cell cloning and clonal expansion, two monoclonals (5F1-R3-3G11 and 16B10-R2-2F1) were selected for further characterization (7).

Supplementary Material 4: Fig. 4. A. Stain-free gels (top) were used to evaluate the binding of anti-TGFα antibodies to the cytotoxic agents DM1, MMAF, and DXd. A change in the migration of the antibody in the gel was observed after coupling. Bottom blots: one hundred nanograms of each ADC and the unconjugated mAbs were loaded in 12% SDS-PAGE gels. The cytotoxic payload bound to the light and heavy chains of the ADCs was analyzed by Western blot using anti-DM1, anti-MMAF, and anti-DXd antibodies, as shown below. B. Detection of DM1 bound to 5F1 or 16B10 mAbs, compared to T-DM1. Equal amounts of the nude or coupled antibodies were used. Blots were analyzed using anti-DM1 antibodies. C. Restoration of 16B10-MMAF sensitivity in a *TGFA*-KO clone. The parental cell line IMIM-PC2, the CRISPR/Cas9 clone #25 and the *TGFA* reconstituted clone #13 were seeded in 6-well plates and treated with 2.5 nM of the ADCs (colored boxes). After 5 days, the effect on cell proliferation was assessed by cell counting. The results obtained are represented as the mean ± SD of the triplicates of an experiment that were repeated three times. **P < 0.01, calculated by Mann-Whitney U test.

Supplementary Material 5: Fig. 5. A. IC_50_ ± SD of the 16B10-MMAF ADC or free drug in each pancreatic cancer cell line, including CRISPR/Cas9 clones. All values were calculated using GraphPad Prism 8 software. B. Graphical representation of the values shown in A.

Supplementary Material 6: Fig. 6. Internalization of anti-TGFα-MMAF antibodies in pancreatic cancer cell lines. Capan-1 cells were treated for 12 hours at 37°C with 2.5 nM of 5F1-MMAF or 16B10-MMAF, with or without prior incubation with 50 µM of chloroquine (Ch), 3 hours before. White dots indicate colocalization between the anti-TGFα ADCs and LAMP1, which is represented by scatter graphs at the bottom. Scale: 25 µm. Anti-TGFα ADCs: red, LAMP1: green; DAPI: blue.

Supplementary Material 7: Fig. 7. A. Expression levels of total and active EGFR pancreatic cancer cell lines. One milligram of total protein from each cell line was immunoprecipitated with an anti-EGFR antibody to assess both the total amount and the phosphorylation status of EGFR. Calnexin was used as a loading control. B. Relationship between *TGFA* and *EGFR* expression in pancreatic cancer. Data was obtained from the TNMplot online tool.

Supplementary Material 8

## Data Availability

No datasets were generated or analysed during the current study.

## Abbreviations

ADCs: Antibody-drug conjugate
BSA: Bovine serum albumin
CCLE: Cancer Cell Line Encyclopedia
CRISPR: Clustered regularly interspaced short palincromic repeats
DAPI: 4’6-diamidino-2-fenilindol
DM1: Emtansine
DMEM: Dulbecco’s modified Eagle’s medium
DXd: Deruxtecan
EDTA: Ethylenediaminetetraacetic acid
EGF: Epidermal growth factor
EGFR: Epidermal growth factor receptor
FBS: Fetal bovine serum
HCl: Hydrochloric acid
HRP: Horseradish peroxidase
KRH: Krebs-Ringer-HEPES buffer
MAPK: Mitogen-activated protein kinase
MMAF: Monomethyl auristatin F
NaCl: Sodium chloride
PBS: Phosphate-buffered saline
PDAC: Pancreatic ductal adenocarcinoma
PDX: Patient-derived xenograft
PEI: Polyethyleneimine
PI3K: Phosphatidyl inositol 3-kinase
PVDF: Polyvinylidene difluoride
RNAi: RNA interference
SDS-PAGE: Sodium dodecyl sulfate polyacrylamide gel electrophoresis
shRNA: short hairpin RNA
TBST: Tris-buffer saline with tween
TCEP: Tris (2-carboxethyl) phosphine
TGFα: Transforming growth factor alpha
