# Supplementary material for: An antibody-drug conjugate targeting soluble and membrane-bound TGFα is effective against pancreatic tumors
Source: J Exp Clin Cancer Res. 2025 May 23;44:158. doi: 10.1186/s13046-025-03421-8 (PMC12100920; doi:10.1186/s13046-025-03421-8)

Supplementary figure 1

A

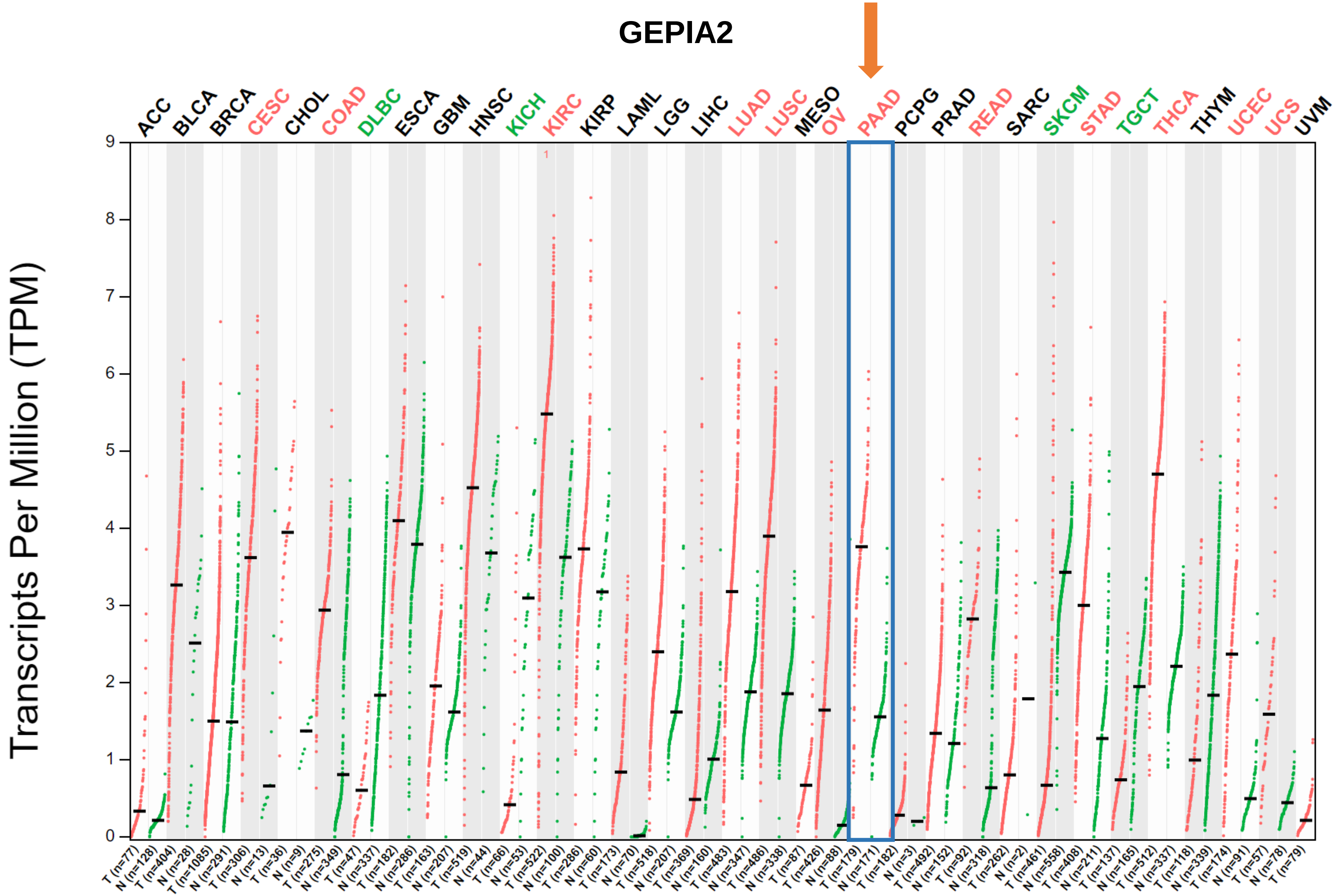

B

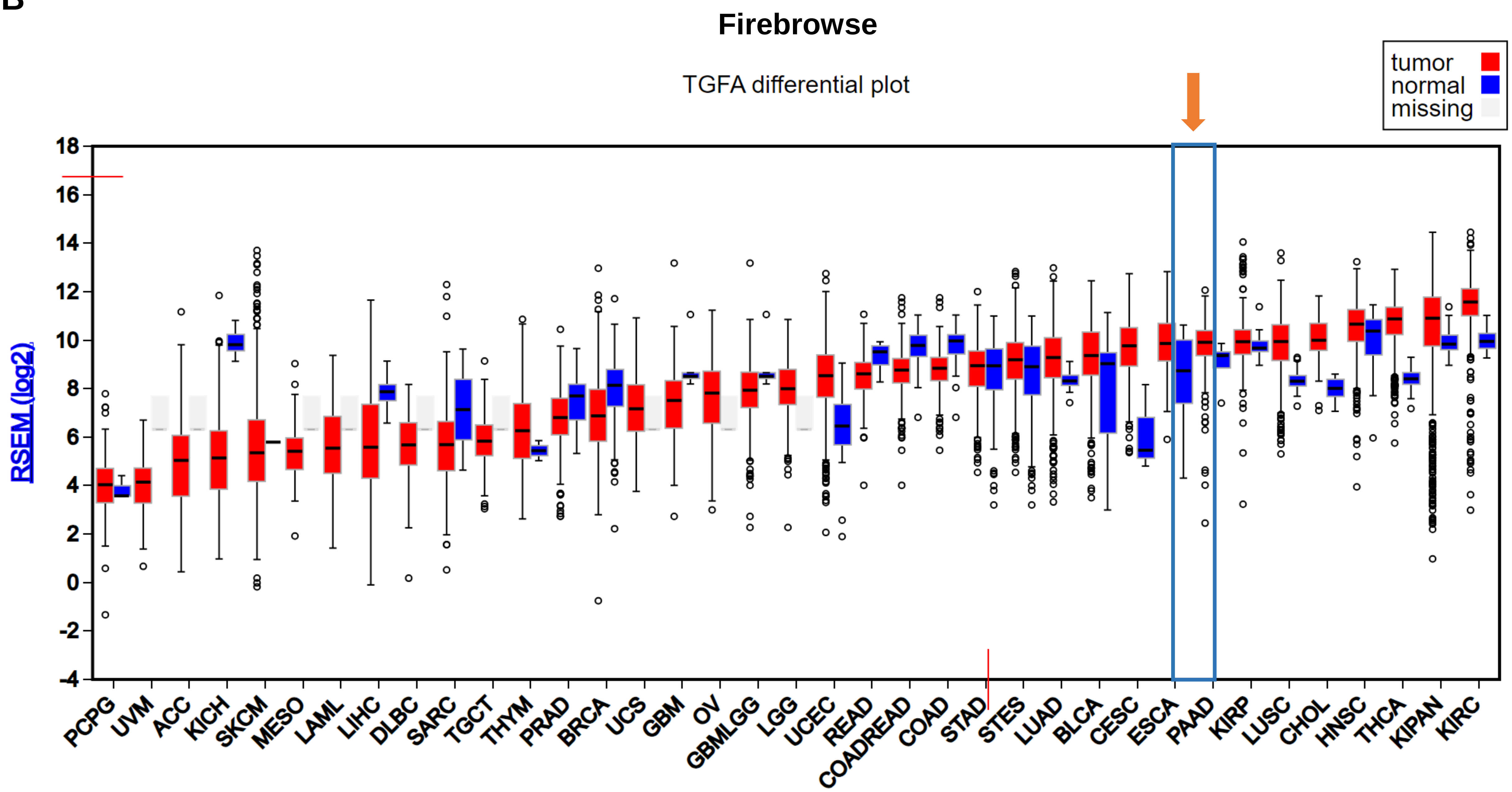
Firebrowse
TGFA differential plot
tumor
normal
missing
RSEM (log2)
18
16
14
12
10
8
6
4
2
0
-2
-4
PCPG
UVM
ACC
KICH
SKCM
MESO
LAML
LIHC
DLBC
SARC
TGCT
THYM
PRAD
BRCA
UCS
GBM
OV
GBMLGG
LGG
UCEC
READ
COADREAD
COAD
STAD
STES
LUAD
BLCA
CESC
ESCA
PAAD
KIRP
LUSC
CHOL
HNSC
THCA
KIPAN
KIRC

C

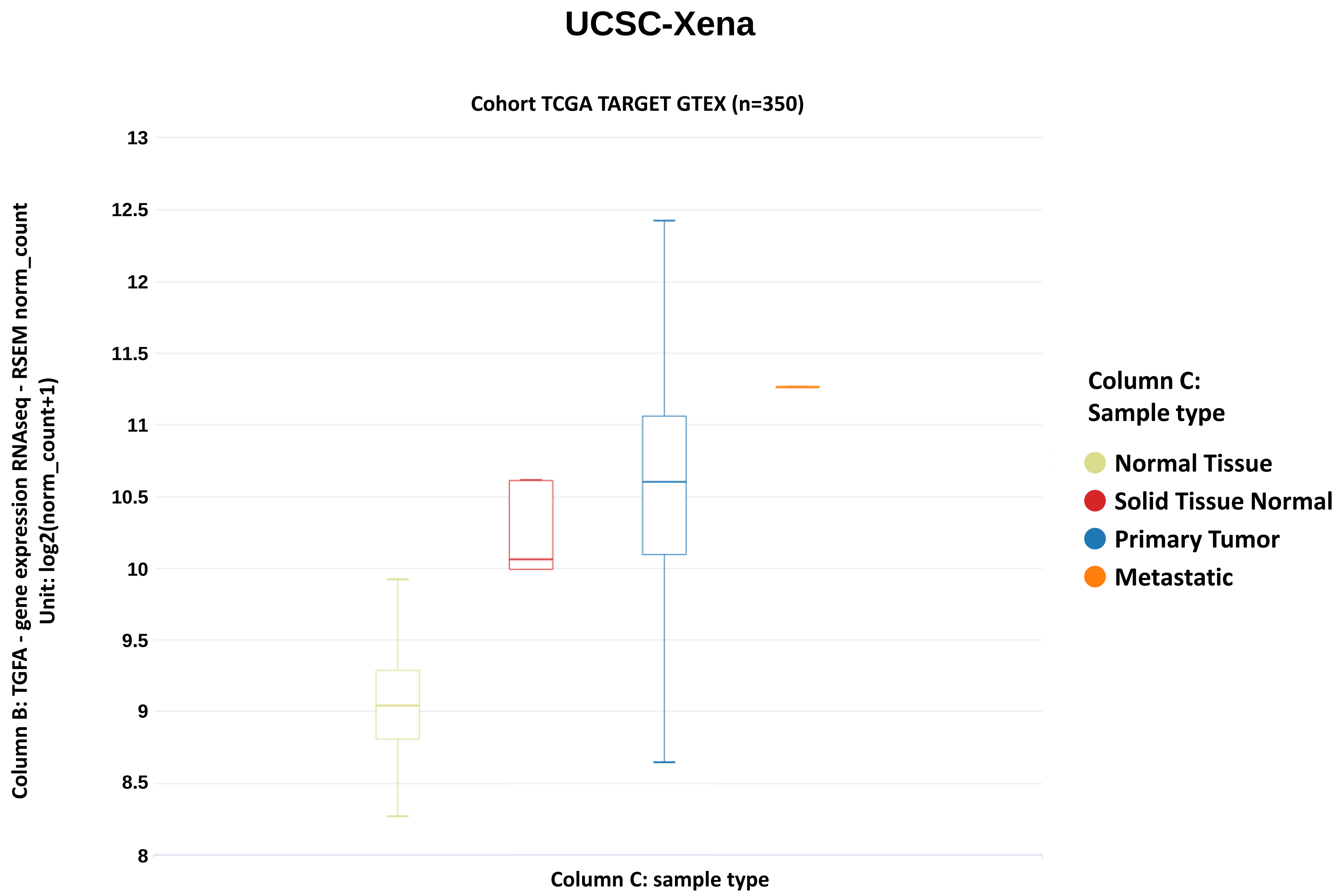
UCSC-Xena
Cohort TCGA TARGET GTEX (n=350)
13
12.5
12
11.5
11
10.5
10
9.5
9
8.5
8
Column B: TGFA - gene expression RNAseq - RSEM norm_count
Unit: log2(norm_count+1)
Column C: sample type
Column C:
Sample type
Normal Tissue
Solid Tissue Normal
Primary Tumor
Metastatic

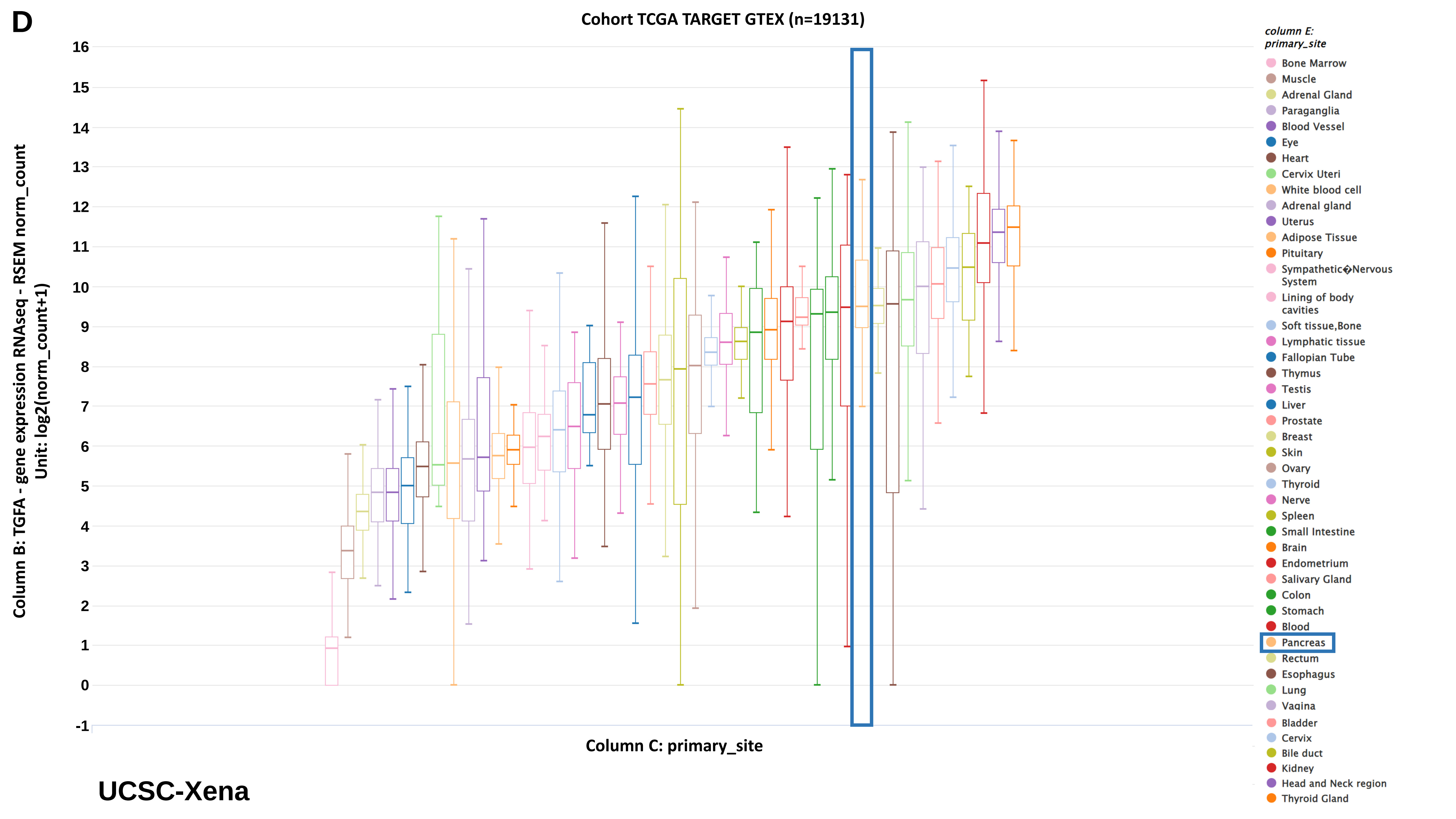
D
Cohort TCGA TARGET GTEX (n=19131)
Column B: TGFA - gene expression RNAseq - RSEM norm_count
Unit: log2(norm_count+1)
16
15
14
13
12
11
10
9
8
7
6
5
4
3
2
1
0
-1
Column C: primary_site
column E: primary_site
Bone Marrow
Muscle
Adrenal Gland
Paraganglia
Blood Vessel
Eye
Heart
Cervix Uteri
White blood cell
Adrenal gland
Uterus
Adipose Tissue
Pituitary
Sympathetic�Nervous System
Lining of body cavities
Soft tissue,Bone
Lymphatic tissue
Fallopian Tube
Thymus
Testis
Liver
Prostate
Breast
Skin
Ovary
Thyroid
Nerve
Spleen
Small Intestine
Brain
Endometrium
Salivary Gland
Colon
Stomach
Blood
Pancreas
Rectum
Esophagus
Lung
Vagina
Bladder
Cervix
Bile duct
Kidney
Head and Neck region
Thyroid Gland
UCSC-Xena

E

cBioPortal

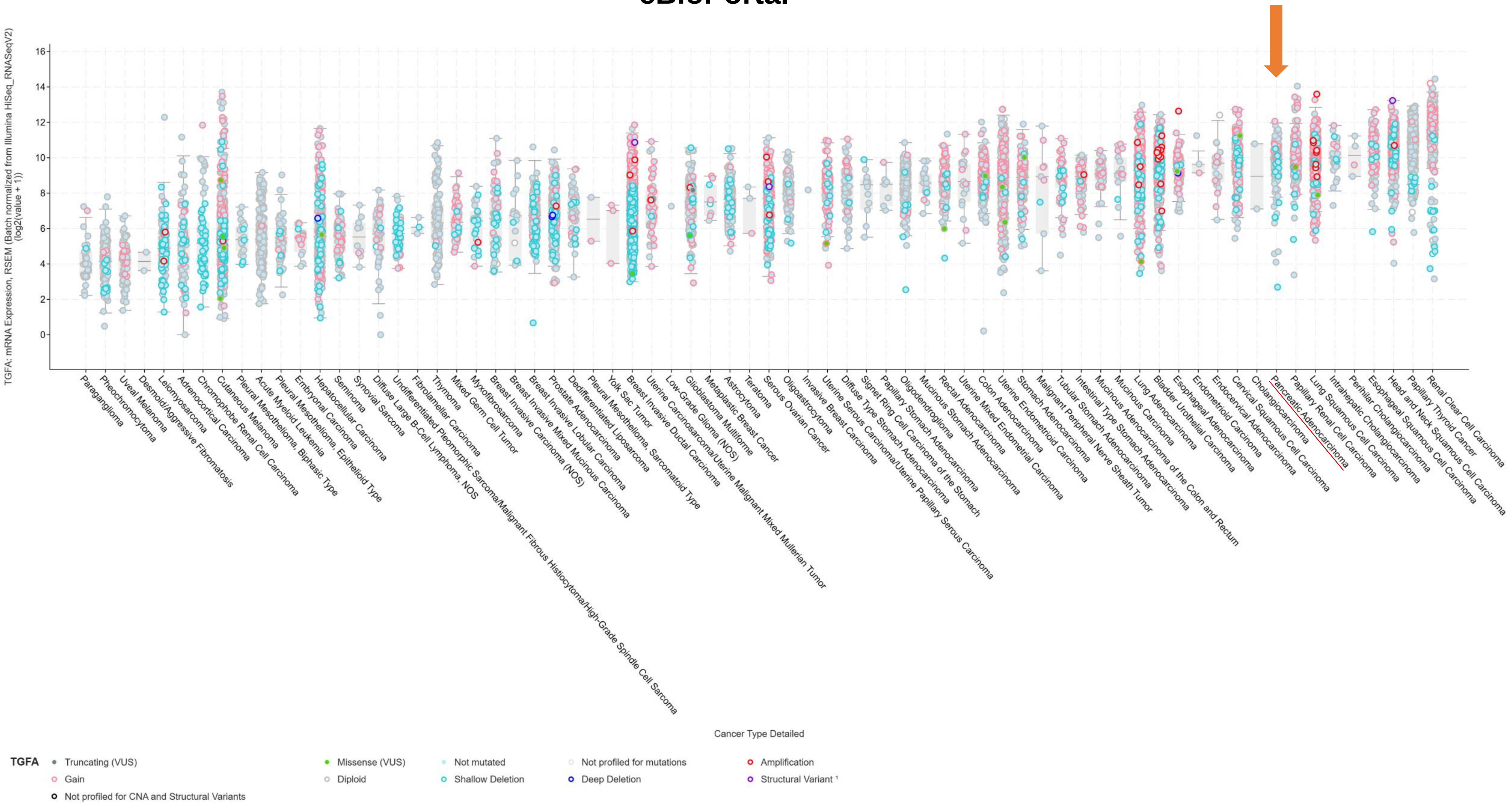

Supplement: Supplementary file 1 — Supplementary Material 1: Fig. 1. A, B. In silico data (from GEPIA2 in A, and Firebrowse in B) on TGFA expression in various tumors, compared to normal tissue. The arrow indicates the pancreatic normal and tumoral tissues, which are also marked by a blue rectangle. In red, the tissues in which TGFA expression is significatively higher in the tumoral vs. normal samples. Abbreviations of the tissues detailed in the respective web pages. C. In silico data (from UCSC-Xena) on TGFA expression in pancreatic tumors (primary site and metastatic), compared to normal tissue. D. Ranking of TGFA expression in different tumors, as obtained from the UCSC-Xena online tool. A blue rectangle marks pancreatic tumors. E. Ranking of TGFA expression in different tumors, as obtained from the cBioPortal online tool. The arrow marks pancreatic tumors. [file 13046_2025_3421_MOESM1_ESM.pdf]
