# Supplementary figures and images for: An antibody-drug conjugate targeting soluble and membrane-bound TGFα is effective against pancreatic tumors
Source: J Exp Clin Cancer Res. 2025 May 23;44:158. doi: 10.1186/s13046-025-03421-8 (PMC12100920; doi:10.1186/s13046-025-03421-8)

Supplementary figure 2

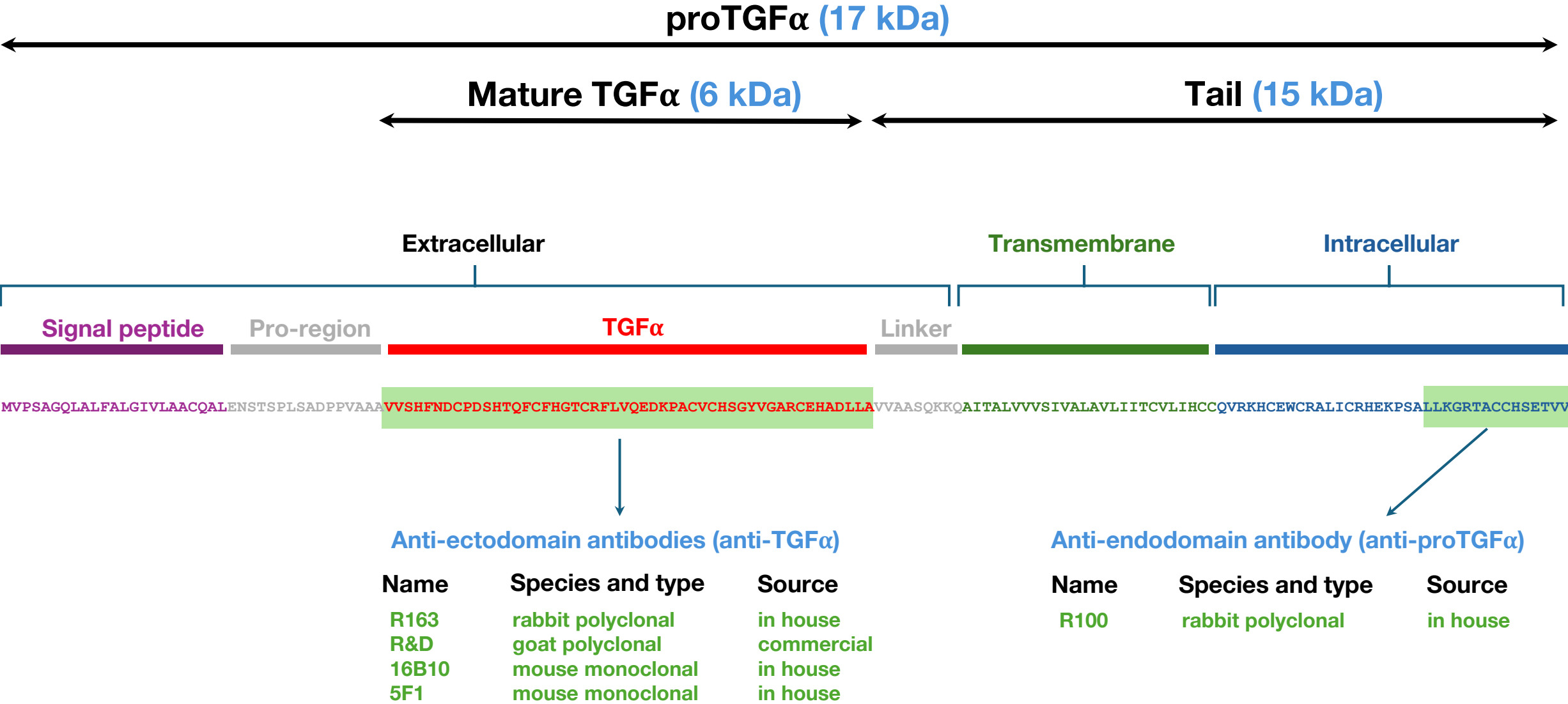

Supplement: Supplementary file 2 — Supplementary Material 2: Fig. 2. Representative diagram of proTGFα domains, indicating the molecular weight corresponding to different forms, the amino acid sequence, as well as the different antibodies generated against this protein (antibody name, species, type, and source). [file 13046_2025_3421_MOESM2_ESM.pdf]

Supplementary figure 4

A

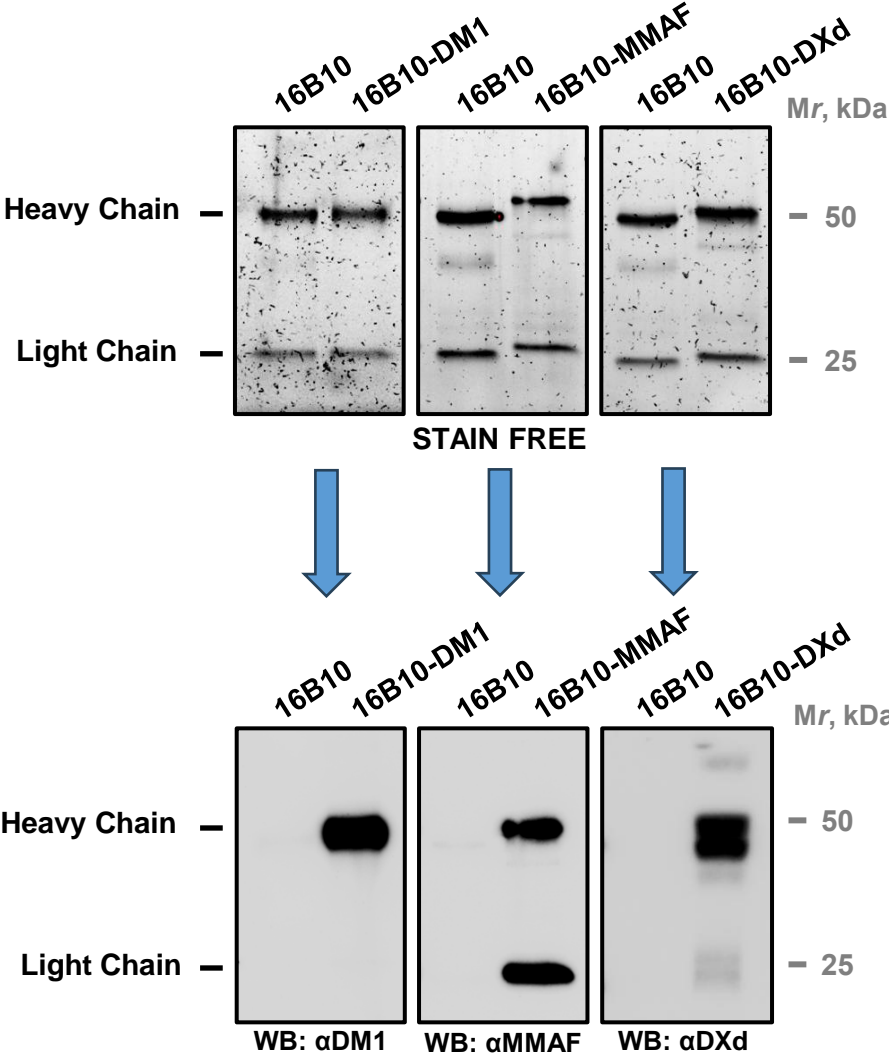

B

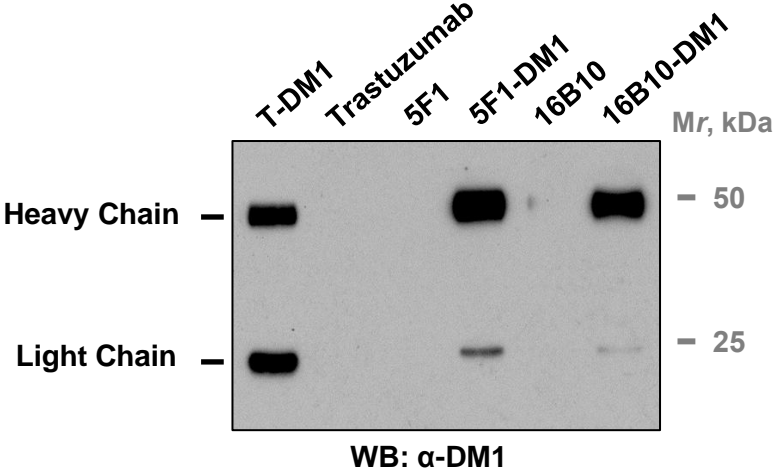

C

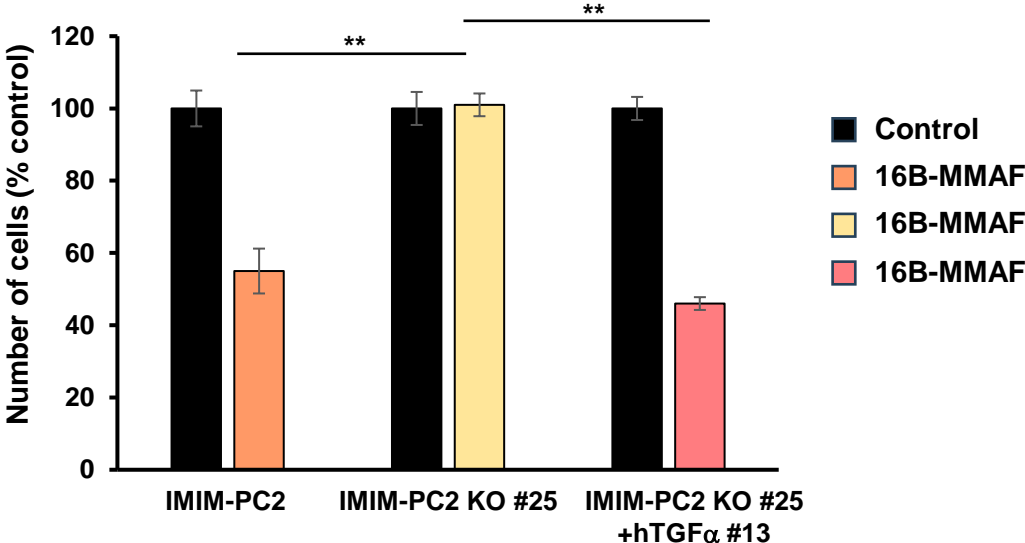

Supplement: Supplementary file 4 — Supplementary Material 4: Fig. 4. A. Stain-free gels (top) were used to evaluate the binding of anti-TGFα antibodies to the cytotoxic agents DM1, MMAF, and DXd. A change in the migration of the antibody in the gel was observed after coupling. Bottom blots: one hundred nanograms of each ADC and the unconjugated mAbs were loaded in 12% SDS-PAGE gels. The cytotoxic payload bound to the light and heavy chains of the ADCs was analyzed by Western blot using anti-DM1, anti-MMAF, and anti-DXd antibodies, as shown below. B. Detection of DM1 bound to 5F1 or 16B10 mAbs, compared to T-DM1. Equal amounts of the nude or coupled antibodies were used. Blots were analyzed using anti-DM1 antibodies. C. Restoration of 16B10-MMAF sensitivity in a TGFA-KO clone. The parental cell line IMIM-PC2, the CRISPR/Cas9 clone #25 and the TGFA reconstituted clone #13 were seeded in 6-well plates and treated with 2.5 nM of the ADCs (colored boxes). After 5 days, the effect on cell proliferation was assessed by cell counting. The results obtained are represented as the mean ± SD of the triplicates of an experiment that were repeated three times. **P < 0.01, calculated by Mann-Whitney U test. [file 13046_2025_3421_MOESM4_ESM.pdf]

## Supplementary figure 6

### Capan-1

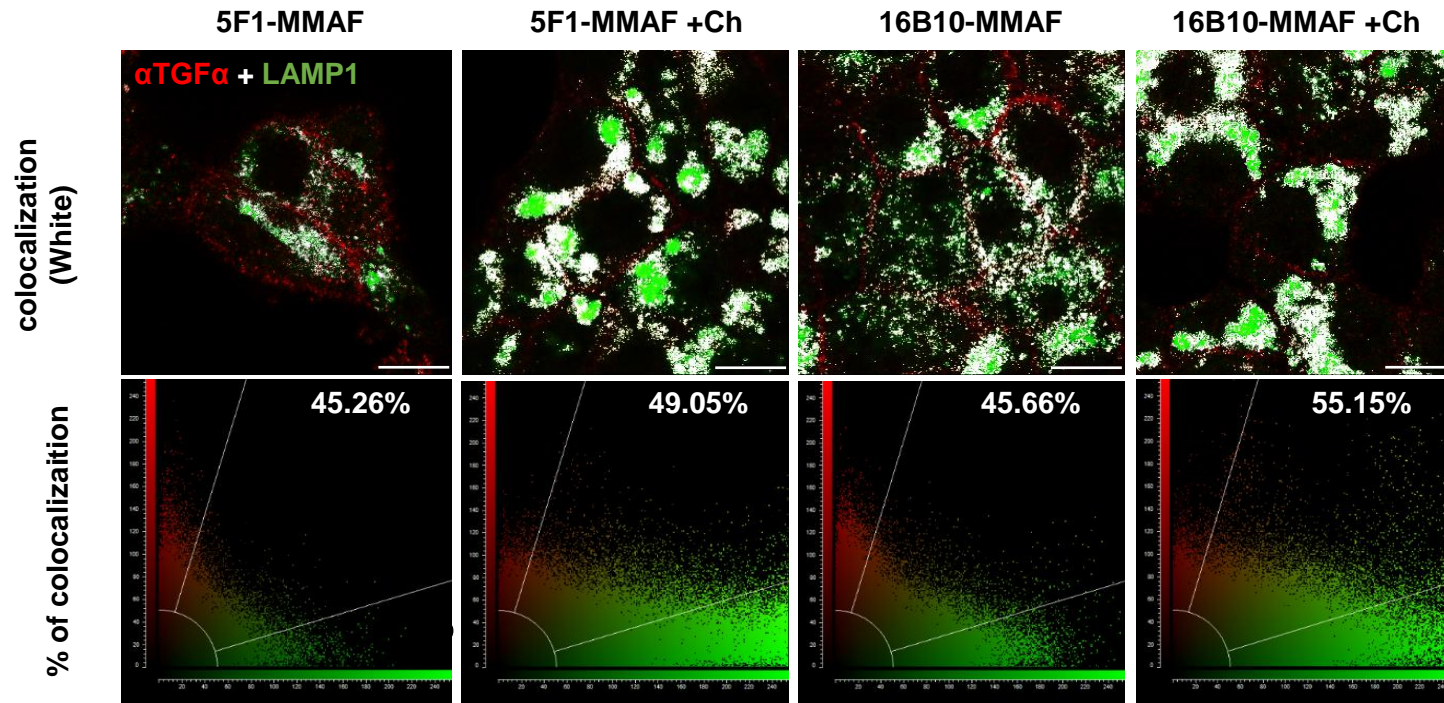

Supplement: Supplementary file 6 — Supplementary Material 6: Fig. 6. Internalization of anti-TGFα-MMAF antibodies in pancreatic cancer cell lines. Capan-1 cells were treated for 12 hours at 37°C with 2.5 nM of 5F1-MMAF or 16B10-MMAF, with or without prior incubation with 50 µM of chloroquine (Ch), 3 hours before. White dots indicate colocalization between the anti-TGFα ADCs and LAMP1, which is represented by scatter graphs at the bottom. Scale: 25 µm. Anti-TGFα ADCs: red, LAMP1: green; DAPI: blue. [file 13046_2025_3421_MOESM6_ESM.pdf]

Supplementary figure 7

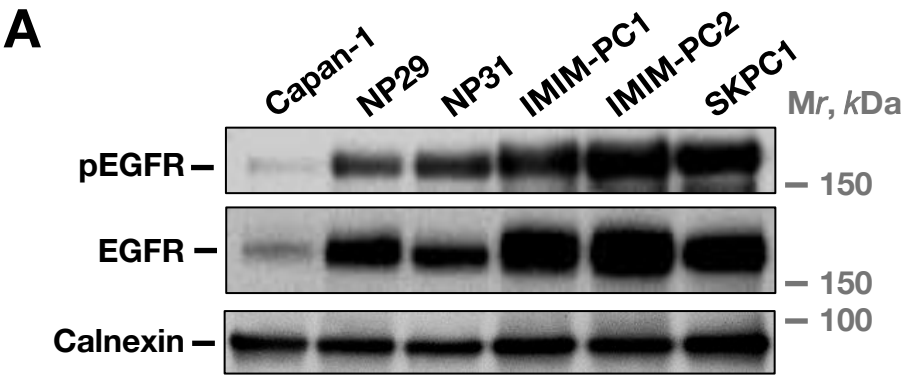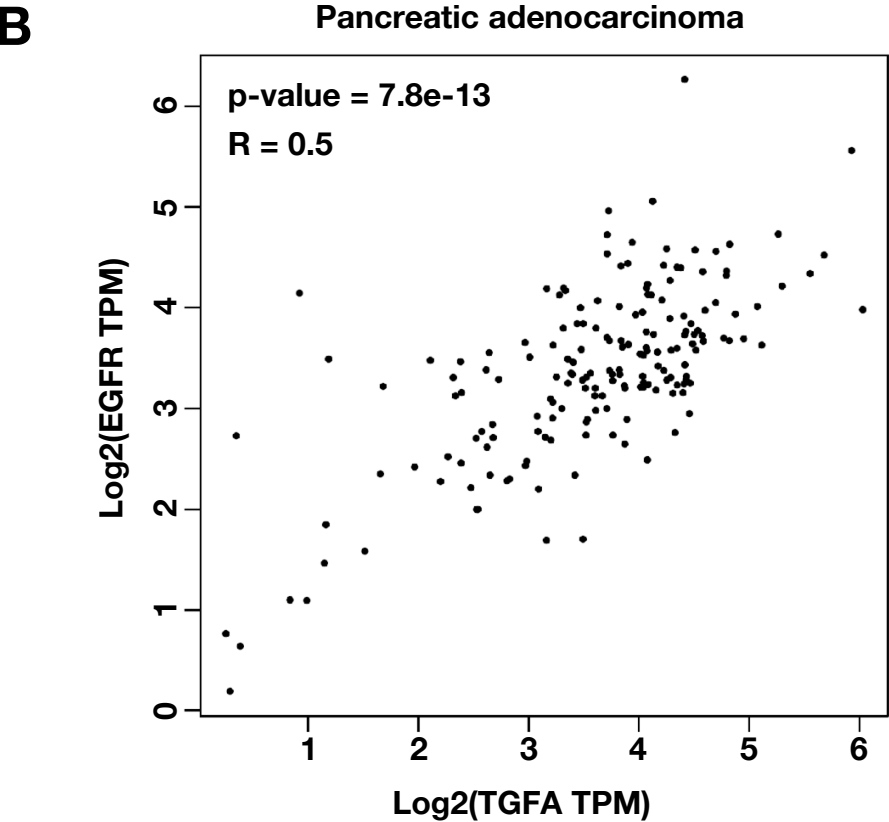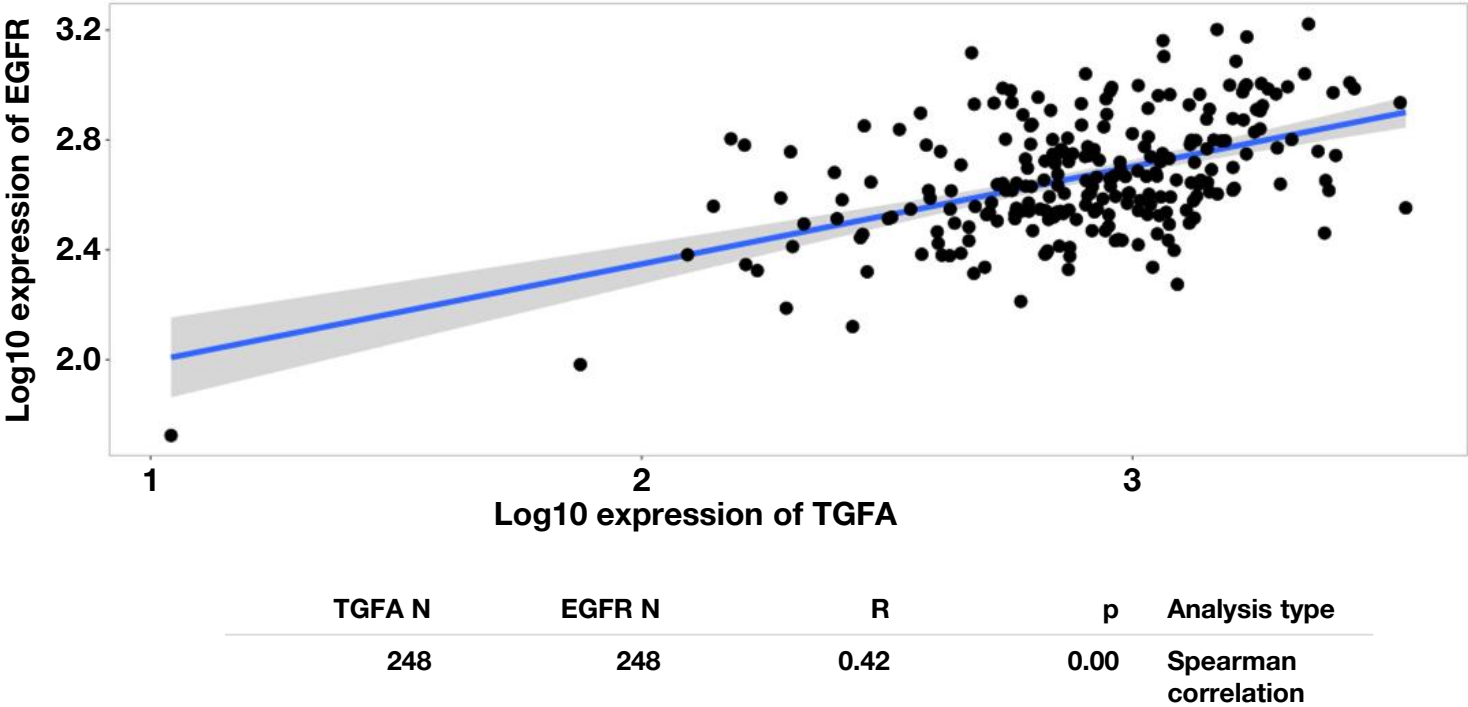

Supplement: Supplementary file 7 — Supplementary Material 7: Fig. 7. A. Expression levels of total and active EGFR pancreatic cancer cell lines. One milligram of total protein from each cell line was immunoprecipitated with an anti-EGFR antibody to assess both the total amount and the phosphorylation status of EGFR. Calnexin was used as a loading control. B. Relationship between TGFA and EGFR expression in pancreatic cancer. Data was obtained from the TNMplot online tool. [file 13046_2025_3421_MOESM7_ESM.pdf]
