# Supplementary material for: An antibody-drug conjugate targeting soluble and membrane-bound TGFα is effective against pancreatic tumors
Source: J Exp Clin Cancer Res. 2025 May 23;44:158. doi: 10.1186/s13046-025-03421-8 (PMC12100920; doi:10.1186/s13046-025-03421-8)

Supplementary figure 3

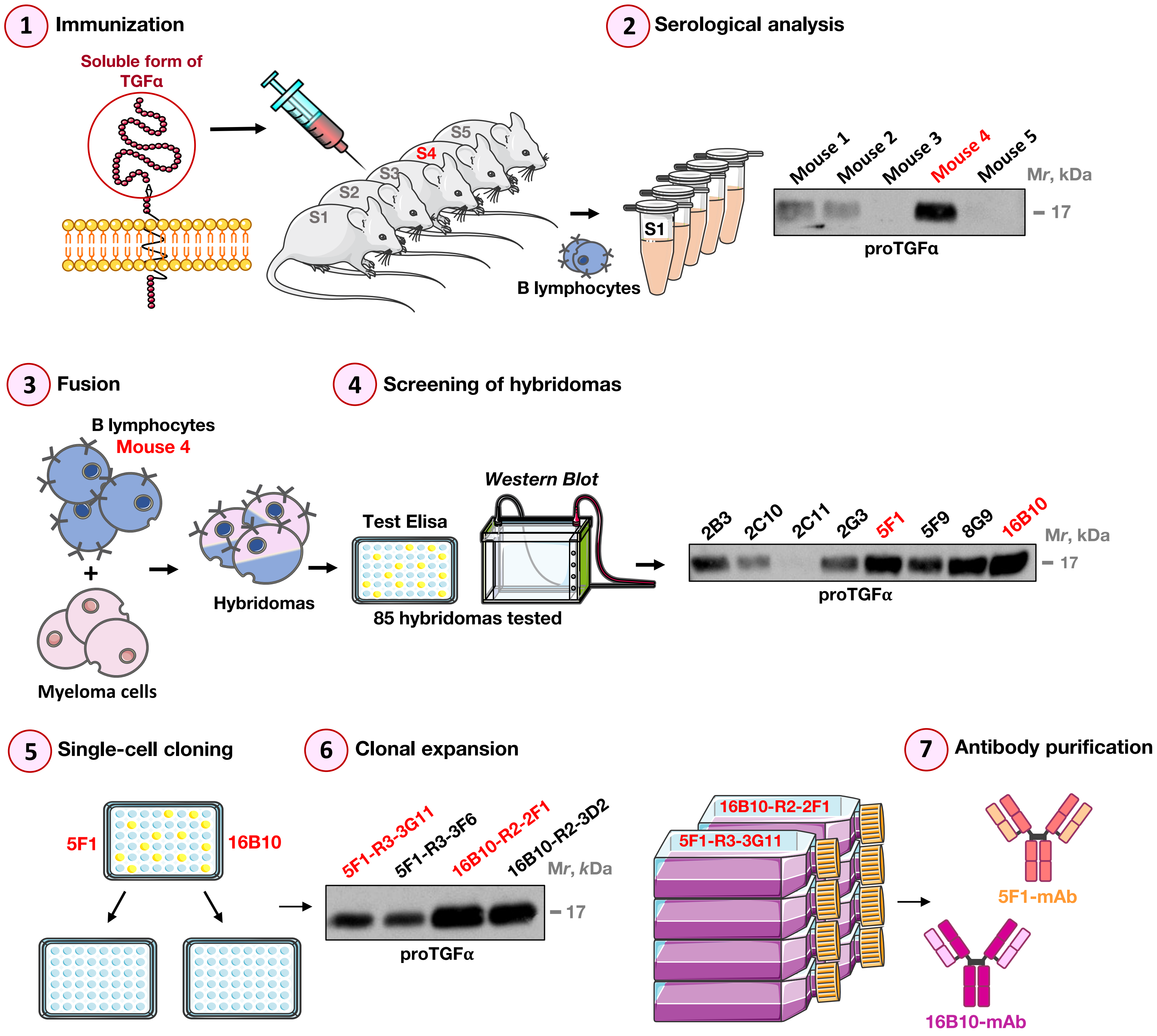

Supplement: Supplementary file 3 — Supplementary Material 3: Fig. 3. Generation of the monoclonal antibodies. After the initial immunization (1), three of the injected mice raised antibodies able to recognize proTGFα when the culture supernatants were tested in immunoprecipitation experiments (2). One of these mice, #4, gave the best results (2) and was then selected to carry out fusions and selection of oligoclonal populations (3). ELISA of mature TGFα used as the screening strategy for the testing culture supernatants from 85 different oligoclonal populations (4), and then positive oligoclonal cultures were used to test them in immunoprecipitation experiments of native proTGFα (4) using extracts of NP29 cells. These studies led to the selection of eight of those populations, which upon a second round of expansion and single cell cloning (5) allowed expansion of four different subclones (6). After single cell cloning and clonal expansion, two monoclonals (5F1-R3-3G11 and 16B10-R2-2F1) were selected for further characterization (7). [file 13046_2025_3421_MOESM3_ESM.pdf]
