# Supplementary material for: An antibody-drug conjugate targeting soluble and membrane-bound TGFα is effective against pancreatic tumors
Source: J Exp Clin Cancer Res. 2025 May 23;44:158. doi: 10.1186/s13046-025-03421-8 (PMC12100920; doi:10.1186/s13046-025-03421-8)

Supplementary figure 5

A

Cell line	16B10-MMAF (IC_{50}, nM)	Free MMAF (IC_{50}, nM)
NP29 KO #8	Not reached	36.2 ± 6.39
NP31 KO #22	Not reached	610.4 ± 228.7
IMIM-PC2 KO #25	Not reached	276.9 ± 205.8
SKPC1	0.05 ± 0.03	81.7 ± 22.9
NP29	0.15 ± 0.04	32.8 ± 4.14
Capan-1	0.32 ± 0.08	51 ± 21.8
NP31	1.06 ± 0.29	658.6 ± 146.2
IMIM-PC2	3.83 ± 1.91	206.7 ± 99.7
IMIM-PC1	9.17 ± 4.24	710.7 ± 83.4

B

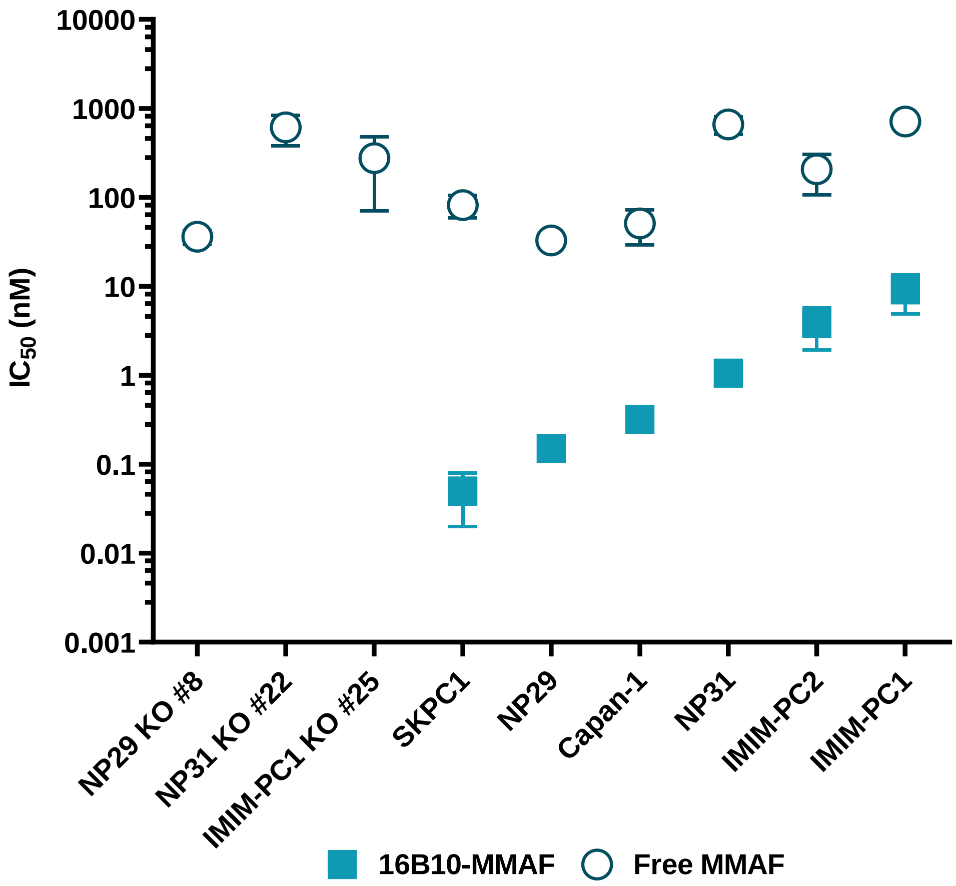

Supplement: Supplementary file 5 — Supplementary Material 5: Fig. 5. A. IC50 ± SD of the 16B10-MMAF ADC or free drug in each pancreatic cancer cell line, including CRISPR/Cas9 clones. All values were calculated using GraphPad Prism 8 software. B. Graphical representation of the values shown in A. [file 13046_2025_3421_MOESM5_ESM.pdf]
