# Supplementary material for: An antibody-drug conjugate targeting soluble and membrane-bound TGFα is effective against pancreatic tumors
Source: J Exp Clin Cancer Res. 2025 May 23;44:158. doi: 10.1186/s13046-025-03421-8 (PMC12100920; doi:10.1186/s13046-025-03421-8)
Supplement: Supplementary file 8 — Supplementary Material 8 [file 13046_2025_3421_MOESM8_ESM.pdf]

Supplementary Table 1. ***KRAS*** **mutation status of the cell lines used in this work**

Cell line	RRID	*KRAS* mutation status and source	
		Cellosaurus*	This study
CAPAN-1	CVCL_0237	G12V (c.35G>T)	Mutation detected (G12X)
IMIM-PC1	CVCL_4061	G12D (c.35G>A)	Mutation detected (G12X)
IMIM-PC2	CVCL_0345	G12D (c.35G>A)	Mutation detected (G12X)
SK-PC-1	CVCL_4054	G12D (c.35G>A)	Mutation detected (G12X)
NP29	CVCL_E3BR	Not available	Mutation detected (G12C)
NP31	CVCL_E3BS	Not available	Mutation detected (G12X)

*https://www.cellosaurus.org
